# The bone microstructure of polar “hypsilophodontid” dinosaurs from Victoria, Australia

**DOI:** 10.1038/s41598-018-19362-6

**Published:** 2018-01-18

**Authors:** Holly N. Woodward, Thomas H. Rich, Patricia Vickers-Rich

**Affiliations:** 1 Oklahoma State University Center for Health Sciences, Tulsa, Oklahoma United States of America; 2 Museums Victoria, Melbourne, Victoria, Australia; 3 Swinburne University of Science and Technology, Melbourne, Victoria, Australia; 4 School of Earth, Atmosphere and Environment, Monash University, Melbourne, Victoria, Australia; 5 Deakin University, Melbourne, Victoria, Australia

## Abstract

High-latitude (i.e., “polar”) Mesozoic fauna endured months of twilight and relatively low mean annual temperatures. Yet non-avian dinosaurs flourished in this taxing environment. Fossils of basal ornithopod dinosaurs (“hypsilophodontids”) are common in the Early Cretaceous high-latitude sediments of Victoria, Australia, and four taxa have been described; although their ontogenetic histories are largely unexplored. In the present study, eighteen tibiae and femora were utilized in the first multi-specimen ontogenetic histological analysis of Australian polar hypsilophodontids. The sample consists of eleven individuals from the Flat Rocks locality (Late Valanginian or Barremian), and five from the Dinosaur Cove locality (Albian). In both groups, growth was most rapid during the first three years, and skeletal maturity occurred between five and seven years. There is a weak asymptotic trend in a plot of growth mark count versus femur length, with considerable individual variation. Histology suggests two genera are present within the Dinosaur Cove sample, but bone microstructure alone could not distinguish genera within the Flat Rocks sample, or across the two geologically separate (~ 26 Ma) localities. Additional histologic sampling, combined with morphological analyses, may facilitate further differentiation between ontogenetic, individual, and species variation.

## Introduction

During the Mesozoic, non-avian dinosaurs radiated to inhabit every continent, even dispersing to paleolatitudes wholly within the Arctic and Antarctic circles^1^. Vertebrates living in these areas today experience prolonged periods of dark or twilight as well as low mean annual temperatures. High-latitude (i.e., “polar”) non-avian dinosaurs, therefore, attract special attention because of the comparatively unique conditions they endured, raising questions concerning possible adaptations and physiologies enabling them to flourish in such environments. To this end, the discipline of paleohistology is often utilized to assess the life histories of polar dinosaurs^2–6^, as examination of bone tissue microstructure reveals age, annual growth rates, and maturity status.

Australia was located within the Antarctic Circle during the Early Cretaceous^7,8^, and basal ornithopod (“hypsilophodontid” *sensu* Norman *et al*.^9^ and Horner *et al*.^10^) fossils are common in sediments of the Early Cretaceous (Valanginian-Albian) Otway Group^11^ along the coast. A previous histologic analysis concluded that hypsilophodontids from Victoria had physiologies similar to lower-latitude relatives based on comparable primary bone tissue organization and the presence of cyclical growth marks (CGMs)^6^. However, ontogenetic life histories of Victoria hypsilophodontids, and the ontogenies of polar dinosaurs in general, remain largely unexplored. And although the physiology of high- and low-latitude hypsilophodontids was similar^6^, there may have been differences in growth rates, asymptotic body size, and longevity that can only be assessed through histological longitudinal analyses. The sample of 19 ornithopod specimens produced for Woodward *et al*.^6^ (most of which are taxonomically unassigned) is the largest thus far histologically examined for any Arctic or Antarctic dinosaur group. The present study differs from Woodward *et al*.^6^ in part because here the bone microstructure of 18 of those hypsilophodontid specimens are described more fully (Table 1). This includes a more thorough description of the hypsilophodontid femur figured in the first Victoria dinosaur histology study^4^ as well as a pathologic specimen previously only described morphologically^12^. In addition, ontogenetic longitudinal growth data is generated for a subset of the sample. This project, therefore, provides the first detailed ontogenetic description and multi-sample ontogenetic growth data for any Antarctic Circle dinosaur taxon.

**Table 1:** Museum Victoria (NMV) hypsilophodontid specimens examined.

NMV Specimen	Locality	Element	Element Length (mm)	Estimated Femur Length (mm)	CGM Count	EFS?	MorphoBank Accession Numbers
P177935	Dinosaur Cove	Right Femur	114; 208		5	Yes	M437320 M437321 M437322 M437323
P180892	Dinosaur Cove	Left Femur	122; 315		8		M437324
P186047.6	Dinosaur Cove	Right Femur	128		6	Yes	M437325
P186047.7	Dinosaur Cove	Left Tibia	185	128	4		M437326
P186326	Dinosaur Cove	Left Femur	185		3		M437327
P186334	Dinosaur Cove	Right Tibia	172	119	4		M437328
P228360	Dinosaur Cove	Right Tibia	200	138.4	7		M437337
P150054	Flat Rocks	Right Femur	145; 155		3		M437319
P199058	Flat Rocks	Left Femur	89; 145		3		M437329
P199133	Flat Rocks	Right Tibia	16	110.7	3		M437330
P208189	Flat Rocks	Right Tibia	188	130.1	2		M437331
P208204	Flat Rocks	Right Tibia	131; 144	99.6	2		M437332
P208336	Flat Rocks	Left Tibia	187; 194	134.2	5		M437318
P208495	Flat Rocks	Left Femur	86; 130		2		M437333
P210062	Flat Rocks	Right Tibia	113; 115	79.6	1		M437334
P216768	Flat Rocks	Right Femur	47		0		M437335
P221151	Flat Rocks	Left Femur	133; 160		5	Yes	M437336
P228434	Flat Rocks	Left Tibia	218	150.8	8	Yes	M437338

If it was necessary to estimate element length, the partial length measurement is reported first, and the estimated total length is reported following a semicolon. In order to plot femora and tibiae on the same graph, tibia lengths were converted to femur lengths using a femur:tibia ratio of 0.69. Cyclical growth mark (CGM) number and presence of an external fundamental system (EFS) is also reported.

Hypsilophodontid taxa from Victoria have been collected primarily from two localities: Flat Rocks and Dinosaur Cove. Sediments from the Flat Rocks locality are presently regarded as Late Valanginian or Barremian in age^11^, and named hypsilophodontid-grade taxa include *Fulgurotherium australe* and *Qantassaurus intrepidus*. The Dinosaur Cove locality is considered Albian in age, and hypsilophodontid taxa include *Fulgurotherium australe*, *Leaellynasaura amicagraphica* and *Atlascopcosaurus loadsi*^7,13,14^. Designated holotypes of the aforementioned taxa consist of teeth and mandible fragments, with the exception of *Leaellynasaura*, which has a partial skull and referred postcrania^7,15^. Agnolin *et al*.^13^ proposed that based on morphology, the holotype material of *Fulgurotherium*, *Qantassaurus*, and *Atlascopcosaurus* are non-diagnostic to the genus level and so are *nomen dubia*, but this remains an ongoing discussion. In addition to documenting ontogenetic growth in polar dinosaurs, the femur and tibia sample from Woodward *et al*.^6^ will be utilized here to independently assess Victoria hypsilophodontid taxonomic diversity: although variation in annual individual growth rates is to be expected^16^, similarly-sized elements with widely varying growth mark counts or primary bone tissue organization may suggest the presence of more than one hypsilophodontid taxon within the sample from Victoria.

## Results

Detailed qualitative bone microstructural descriptions of each thin section slide can be found in Supplementary Information online, and a summary of the bone tissue microstructures observed in the Flat Rocks and Dinosaur Cove samples follows here. For all hypsilophodontid specimens, the medullary cavity is free of trabeculae and there is very little secondary tissue, even in skeletally mature individuals. In general, primary tissue of hypsilophodontid femora and tibiae are fibro-lamellar or poorly organized parallel-fibred (i.e., loosely parallel-fibred *sensu* Woodward *et al*.^17^) in zones between the innermost three cyclical growth marks (CGMs). In the zones that follow, tissue organization is poorly organized parallel-fibred, or parallel-fibred. In addition, there is no pattern with regard to whether the CGMs present are lines of arrested growth (LAGs) or annuli: some specimens exhibit both kinds of CGMs, while the cortex of others consist exclusively of LAGs.

**Figure 1:**
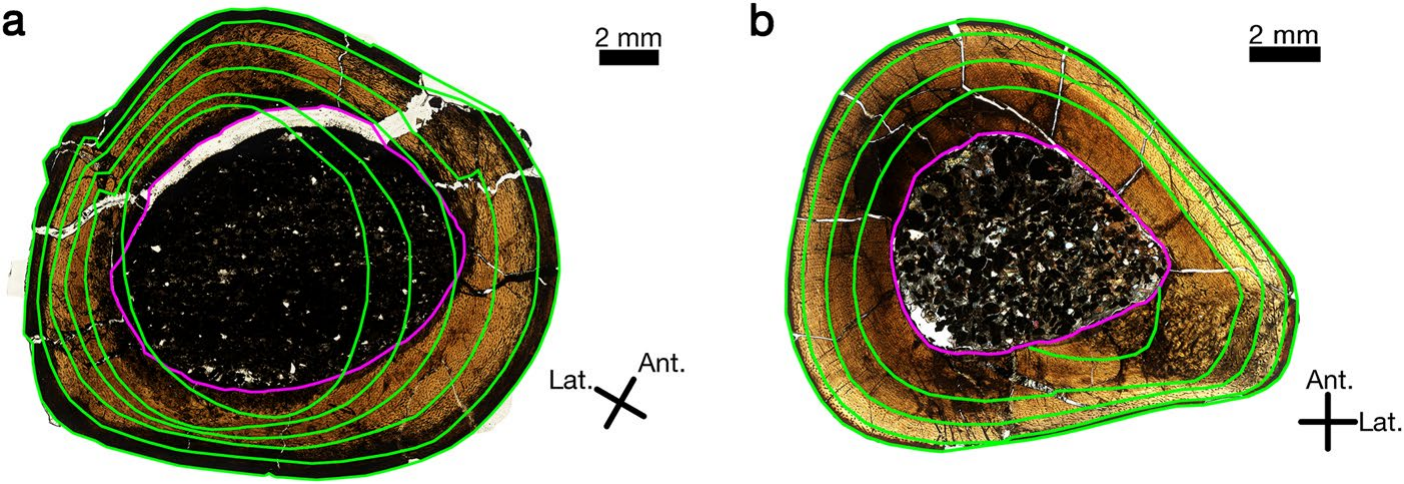
Medullary drift comparison between a hypsilophodontid femur (NMV P221151) and tibia (NMV P186334). (**a**) In hypsilophodontid femora, there was pronounced ontogenetic change in transverse diaphyseal shape, as indicated here by the green rings which trace the outlines of annual CGMs and the periosteal surface. The border of the medullary cavity is traced in pink to illustrate directional medullary drift, which partially obliterated the first three CGMs of NMV P22151. (**b**) Compared to the transverse diaphyseal shape of the femur NMV P22151, the tibia NMV P186334 underwent less annual ontogenetic shape change. The green rings trace the outlines of annual CGMs and the periosteal surface, while the outline of the medullary cavity is traced in pink. Medullary cavity enlargement is less directional compared to that of the femur, and only partially obliterates a single CGM in this case.

**Figure 2:**
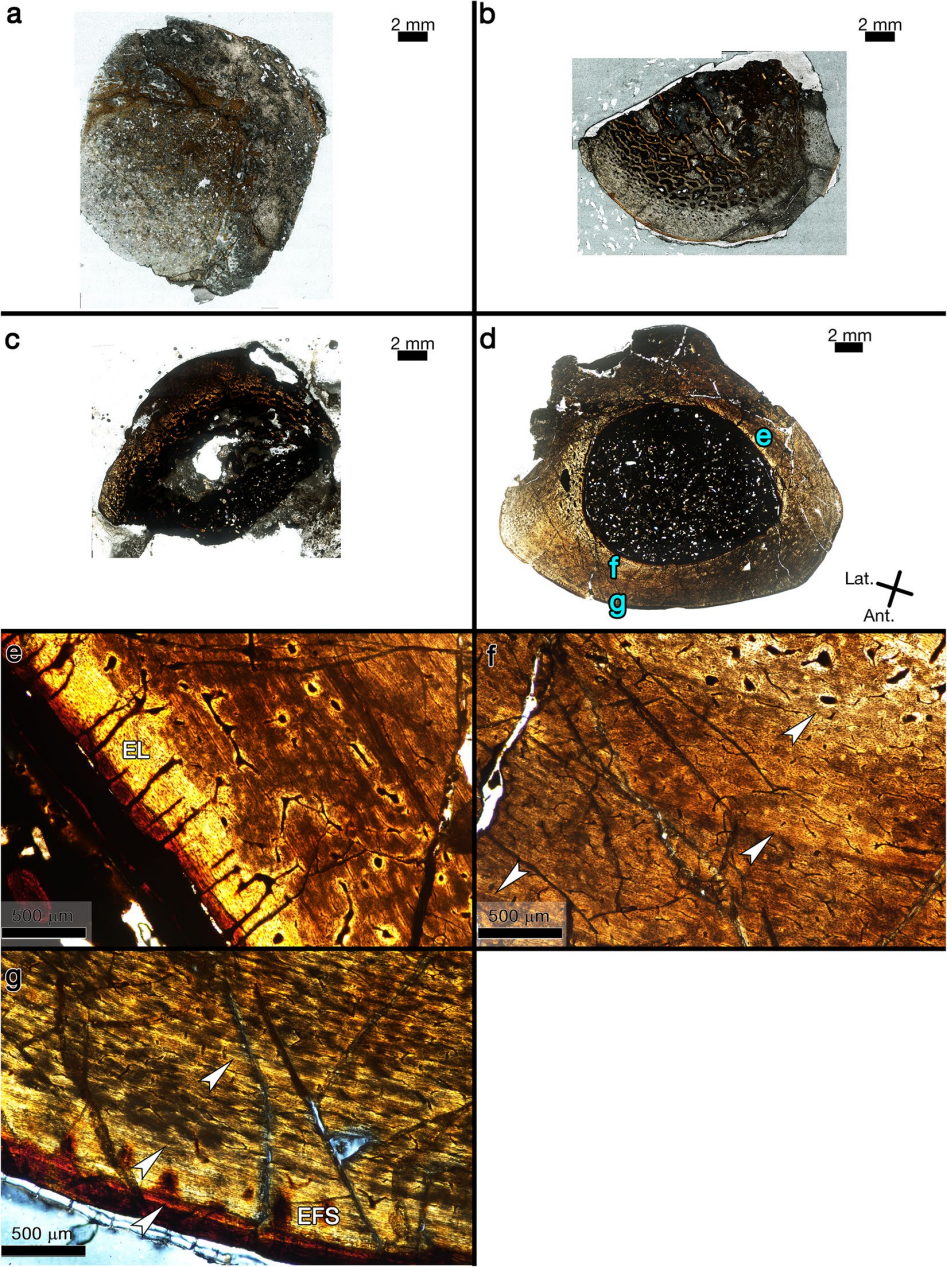
Images of transverse sections from hypsilophodontid right femur NMV P177935. (**a**) Thin section slide designated AI, the first of three transverse thin sections made for the study by Chinsamy *et al*.^4^. Possibly a fragment from the medial side. The thin section was polished too thinly to detect the presence of CGMs. Plane polarized light. (**b**) Thin section slide designated AII, the second of three thin sections made for the study by Chinsamy *et al*.^4^. Possibly a fragment from the anterolateral side. The thin section was polished too thinly to detect the presence of CGMs. Plane polarized light. (**c**) Thin section slide designated AIII, the third of three thin sections made for the study by Chinsamy *et al*.^4^. The thin section was too opaque to detect the presence of CGMs. Plane polarized light. (**d**) Complete transverse section made for the study by Woodward *et al*.^6^. Blue letters reference the magnified regions shown in corresponding panels. Plane polarized light. (**e**) The lamellar endosteal layer (EL) completely encircles the medullary cavity and is frequently intersected by radiating channels. Plane polarized light. (**f**) Compact coarse cancellous bone is visible in the upper right of the panel, and three annuli are visible (arrows) within the cortex. Plane polarized light. (**g**) The third annulus (arrow) is visible within poorly organized parallel-fibred bone, and is followed by two LAGs (arrows) within the well-organised lamellar tissue of an external fundamental system (EFS). Circularly polarized light.

Hypsilophodontid femora and tibiae underwent ontogenetic changes in cortical shape as revealed by medullary drift and directional shifts in cortical apposition rates (Fig. 1). This frequently resulted in partial obliteration of the innermost CGMs. Retrocalculation^18–20^ suggests that three Dinosaur Cove femora (NMV P177935, NMV P186326, and NMV P1808) within the sample had CGMs completely lost to medullary expansion. Overall, directional medullary drift appears more extreme in femora than tibiae (Fig. 1). This drift caused erosion into the primary bone of the innermost cortex. Those regions of the innermost cortex unaffected by drift often consisted of compact coarse cancellous bone (CCCB)^21^, which in many instances was separated from the medullary cavity by a lamellar endosteal layer. Directional medullary drift in femora was in general located on the anterior and lateral sides, so that CCCB was found primarily on the posterior and medial sides. In most, but not all samples, radiating channels were observed traversing the lamellar endosteal bone bounding medullary cavities (e.g., Fig. 2e and Supplementary Figures).

**Figure 3:**
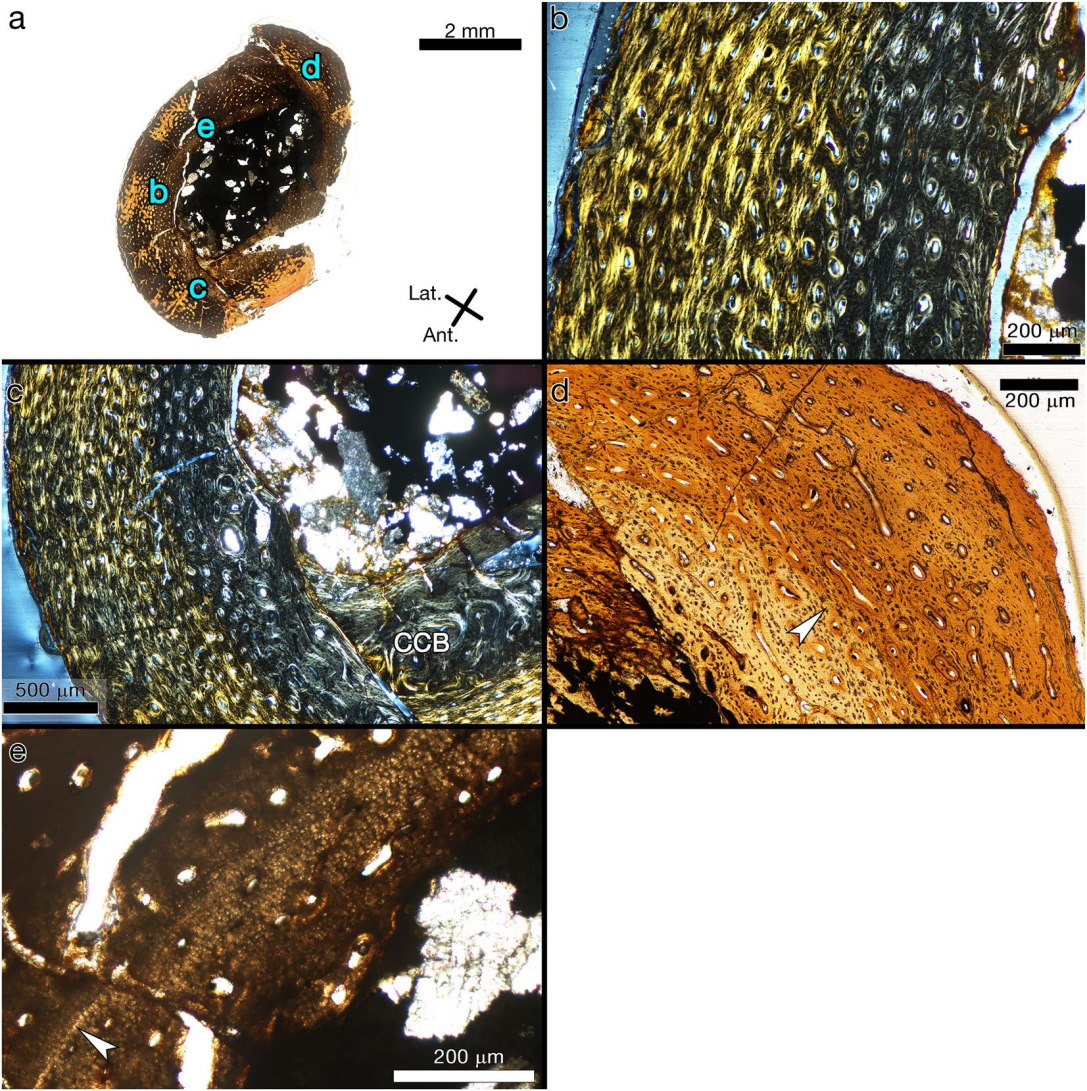
Transverse section images of right ornithopod femur NMV P216768. (**a**) Composite of complete thin section. Blue letters reference the magnified regions shown in corresponding panels. There is a colour change in the tissue mid-cortex (arrow). Plane polarized light. (**b**) Tissue within the lighter coloured inner cortex is primarily fibro-lamellar, while tissue in the darker middle and outer cortex is poorly organized parallel-fibred. Circularly polarized light. (**c**) Compact coarse cancellous bone makes up the majority of the inner cortex on the anterior side. Circularly polarized light. CCB = compact coarse cancellous bone. (**d**) The majority of primary osteons are acellular and resemble the incipient primary osteons described in alligator bone. The boundary of the abrupt colour change (arrow) on the posterior side corresponds to a change in tissue arrangement and may be a neonatal line. Plane polarized light. (**e**) The abrupt colour change on the posterolateral side is demarcated by a birefringent line (arrow). The lighter, innermost cortex is calcified cartilage (upper right of image), indicated by the “bubbly” appearance of the chondrocyte lacunae. Plane polarized light.

The ontogenetically youngest (and also smallest) specimens in the sample are tibiae and femora from the Flat Rocks locality. Two tibiae, NMV P210062 and NMV P208204, have CGM counts of 1 and 2, respectively. Two femora, NMV P216768 and NMV P208495, have counts of 0 and 1, respectively. In addition, the abrupt transition from fibro-lamellar to parallel-fibred tissue in the small femur NMV P216768 resembles the structure referred to as a neonatal “hatching line” described in a juvenile sauropod^22^ (Fig. 3).

The ontogenetically oldest Flat Rocks individual is represented by a tibia (NMV P228434) with reticular parallel-fibred tissue and seven CGMs. There is at least one more (not fully traceable) CGM within the external fundamental system (EFS). An EFS is a region within the outer cortex that is largely avascular, parallel-fibred or lamellar, and may contain LAGs. The presence of an EFS in a femur or tibia indicates the cessation of appreciable increase in body length (i.e., skeletal maturity)^23–26^. Of the eleven Flat Rocks samples, two have EFS: skeletal maturity was achieved at five years for NMV P221151, with a tibia length of 21.9 cm, and at seven years for NMV P228434, with a tibia length of 21.8 cm.

The ontogenetically youngest Dinosaur Cove hypsilophodontids each have four CGMs. Tibia NMV P186334 consists of incipient fibro-lamellar^27,28^ to parallel-fibred tissue with reticular vascular canals. Femur NMV P186326 has three CGMs within poorly organized parallel-fibred tissue and anastomosing to longitudinal simple vascular canals. Qualitative retrocalculation suggests that an innermost CGM was lost to medullary expansion. The oldest Dinosaur Cove specimen is a femur (NMV P180892) with an estimated length of 31.5 cm, having at least 8 CGMs within reticular parallel-fibred tissue. It lacks an EFS, so the individual was still growing prior to death. This particular femur is much larger than the rest of the sample, and the medullary cavity circumference is so large (5.2 cm) that estimation of missing CGMs was not attempted. Two of the Dinosaur Cove specimens have EFS and were skeletally mature in five years with femur lengths of 20.8 cm (NMV P177935; estimated) and 12.8 cm (NMV P186047).

Although detailed histological descriptions are within Supplementary Information online, there are a few specimens warranting special attention and are fully described below.

### NMV P177935

Regional histology of this transverse femur section was figured and briefly described by Chinsamy *et al*.^4^ For that report a total of three slides (labelled AI, AII, and AIII) were produced, together making up the full transverse section of the femur (Fig. 2a–c). Upon examination here, it was discovered that thin section slide AII (Fig. 2c) is too opaque for bone microstructure observation due to the thickness of the slide. Conversely, thin section sides AI and AII were polished so thin that very little of the bone tissue organizational structure remains discernible, including CGMs (Fig. 2a,b). Whole images of the three slides from NMV P177935 were not published with Chinsamy *et al*.^4^, but they are figured here (Fig. 2a–c) and available on Morphobank.org (project P2625).

A second transverse sample from the diaphysis of NMV P177935 was removed and processed by Woodward *et al*.^6^ as part of a study testing the hypothesis proposed by Chinsamy *et al*.^4^ that polar hypsilophodontids lacked CGMs. The thin section slide produced for Woodward *et al*.^6^ comprised the complete transverse section of the diaphysis (Fig. 2d) and they reported the presence of two CGMs within its cortex, thereby rejecting the hypothesis of Chinsamy *et al*.^4^ The histology of NMV P177935 was not described in detail by Woodward *et al*.^6^, so it is discussed here. The transverse section consists of poorly organized parallel-fibred tissue from inner to outer cortex with anastomosing and longitudinal simple vascular canals (Fig. 2e-g). A lamellar endosteal layer completely encircles the medullary cavity. This lamellar endosteal layer is frequently intersected by radial channels (Fig. 2e). The deep innermost cortex on the anterior side consists of compact coarse cancellous bone (Fig. 2f). Flattened osteocyte lacunae are frequent throughout the cortex and arranged in parallel. A total of five CGMs are visible within the primary cortex (Fig. 2f,g), three of which went unnoticed by Woodward *et al*.^6^: two annuli are visible in mid-cortex and one in the outer cortex, and two LAGs are within the region of the EFS which is made of mostly avascular parallel-fibred to lamellar tissue (Fig. 2g). The first of the three annuli is partially obliterated due to medullary cavity expansion and drift. The medullary cavity circumference (4.3 cm) is large enough that, based on LAG circumferences of smaller femora from the dataset, potentially two CGMs are completely obliterated by medullary expansion. If so, the total CGM count would be seven.

### NMV P186047

**Figure 4:**
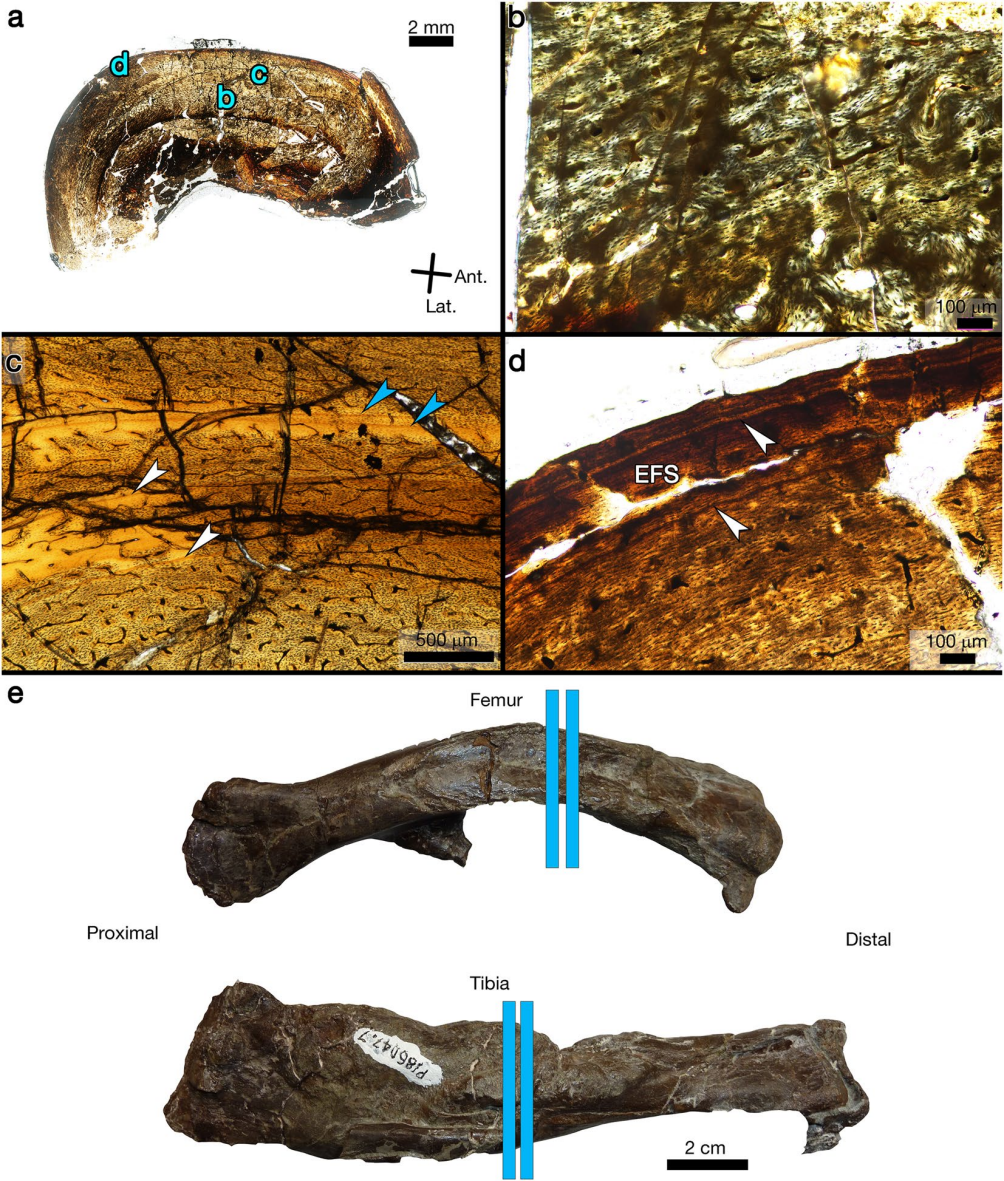
Transverse thin section images of the right femur from NMV P186047, and hand sample images of the right femur and left tibia of NMV P186047. (**a**) Composite image of complete femur thin section. Although the specimen is badly crushed, microstructures are still visible for descriptive purposes. Blue letters reference the magnified regions shown in corresponding panels. Plane polarized light. (**b**) A small region of incipient fibro-lamellar bone is present on the anterior and medial sides of the innermost cortex. Otherwise, bone fibre orientation throughout the cortex is parallel-fibred with longitudinal or anastomosing simple vascular canals. Circularly polarized light. (**c**) Four LAGs are visible within the inner to mid-cortex (arrows). The two LAGs within the annulus (blue arrows) do not merge, and so are considered to represent the growth hiatus of two years. Plane polarized light. (**d**) Bone fibre orientation is more avascular and increasingly lamellar near the periosteal surface, forming an EFS. There is one LAG at the onset of the EFS and another present within the EFS (arrows). Plane polarized light. EFS = external fundamental system. (**e**) Photographs of the right, non-pathologic femur and the left pathologic tibia of NMV P186047. Blue lines show locations of transverse thin section slides produced from the fossils.

This specimen includes an articulated right femur and tibia pair, and a pathologic left tibia. The surface morphology of the left tibia has previously been described in detail^12^ due to its pathologic nature. Despite the post-burial diaphyseal crushing (Fig. 4a), there is no indication that the right femur of this individual suffered from the same pathological condition as the contralateral tibia. Concerning the microstructure of the femur, the innermost cortex of the anteromedial side is compact coarse cancellous bone, separated from the medullary cavity by lamellar endosteal tissue. In this area, radial channels are frequent and travel between the compact coarse cancellous bone and the medullary cavity, through the lamellar endosteal layer. Scalloped primary bone near the medullary cavity on the medial side indicates active resorption prior to death. Except for a small area of incipient fibro-lamellar tissue (*sensu* Klein^27^ and Konietzkoo-Meier and Klein^28^) with small longitudinal primary osteons on the anterior and medial sides (Fig. 4b), the innermost to mid-cortex is poorly organized parallel-fibred tissue with simple longitudinal vascular canals. From the middle to outer cortex, tissue organization is parallel-fibred with anastomosing vascular canals. Osteocyte lacunae are frequent throughout the cortex. There are four LAGs within the mid-cortex (Fig. 4c). Two of these are closely spaced and within an annulus (Fig. 4c, blue arrows). Although they are closely spaced, the two LAGs remain separate when followed around the cortex, so they are considered as representing the growth hiatus of two separate years^17,29^. There are two LAGs in the outermost cortex, one within a thin EFS (Fig. 4d).

**Figure 5:**
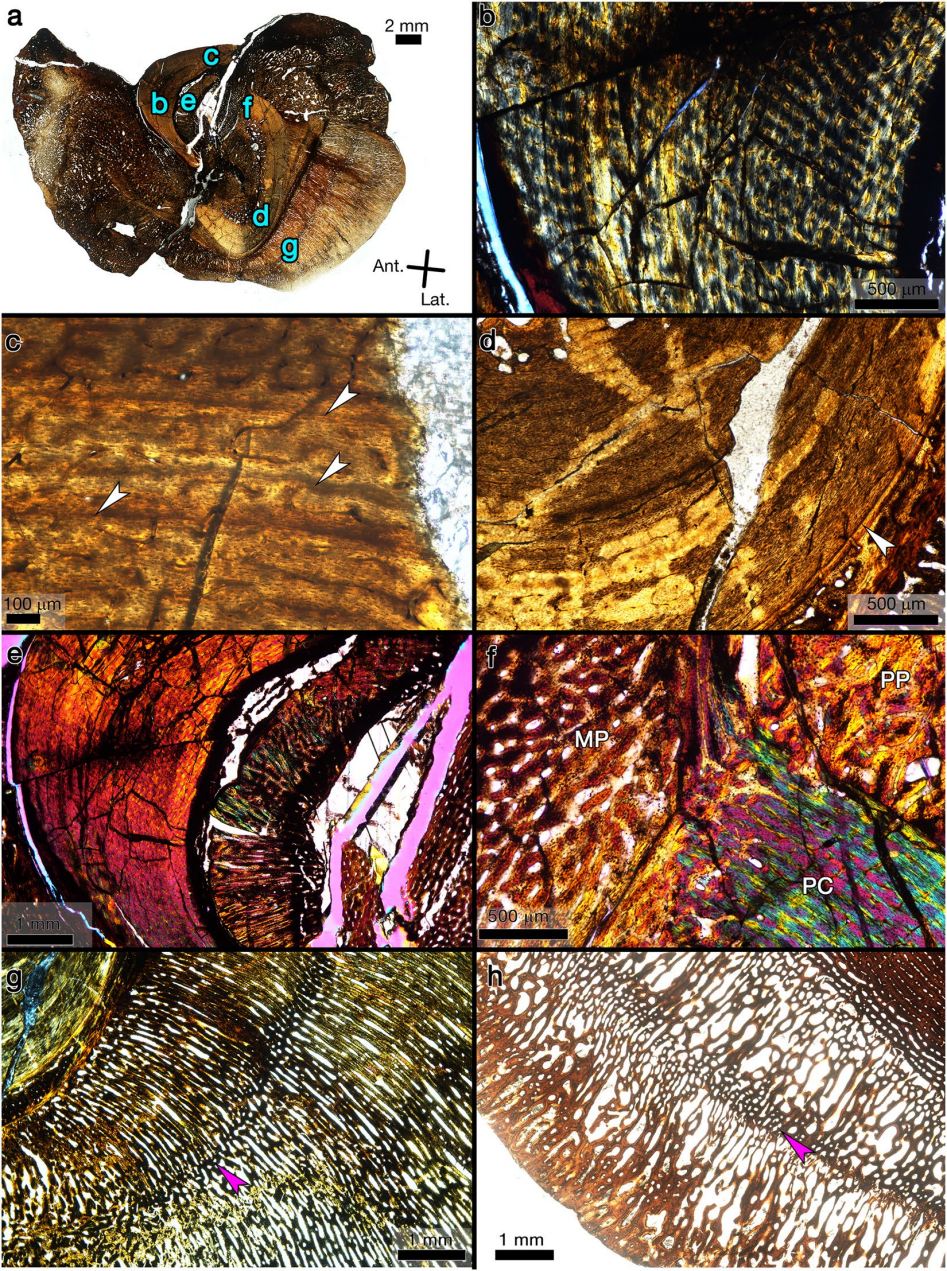
Transverse thin section of left tibia from NMV P186047. The tibia microstructure reveals pathologic outgrowth, previously reported to be the result of osteomyelitis. Blue letters reference the magnified regions shown in corresponding panels. Quarter wave plate. (**b**) The innermost primary cortex varies between fibro-lamellar and poorly organized parallel-fibred with longitudinal primary osteons. The middle to outer cortex is poorly organized parallel-fibred tissue. Vascularity varies from simple primary longitudinal canals, anastomosing canals, longitudinal primary osteons, reticular primary osteons, and radial primary osteons, depending on orientation. Circularly polarized light. (**c**) Three LAGs (arrows) are visible within the mid-cortex. Plane polarized light. (**d**) A fourth LAG (arrow) is located very close to the non-pathologic periosteal surface, and in fact blends into it when traced moving from lateral to medial. Plane polarized light. (**e**) The medullary cavity on the anterior side is filled by woven bone with radial vascularity. Full wave plate. (**f**) The medullary pathologic tissue joins with the pathologic periosteal surface tissue at the opening between the two sheared halves of the primary bone diaphysis, and the pathologic bone blends with the broken surface of the primary diaphyseal bone. Full wave plate. PC = primary cortex; MP = medullary pathologic bone; PP = periosteal surface pathologic bone. (**g**) Detail of periosteal pathologic outgrowth. Vascularity is primarily radial, except in narrow bands (arrow) where it becomes more longitudinal. Circularly polarized light. h) Detail of a laterally directed periosteal outgrowth in the tibia of an immature hadrosaurid, *Maiasaura* (Museum of the Rockies specimen number MOR 005 T-09). This outgrowth is similar in morphology to that observed in the tibia of NMV P186047, consisting of fibro-lamellar tissue with radial vascular canals, interrupted by a “band” of longitudinal fibro-lamellar tissue (arrow). In this image, primary cortex is to the upper right, and the periosteal surface is to the left. Plane polarized light.

The left tibia of NMV P186047 is almost wholly encased within pathologic outgrowth. This outgrowth, in places 10 mm thick^12^, displaced the adjacent fibula, causing it to bow out laterally (Fig. 4e and see Figs 3–5 in Gross *et al*.^12^). In addition, this tibia is 27 mm shorter than the non-pathologic right tibia, presumably from delayed growth due to the pathology^12^. In hand sample, there is one location along the medial mid-diaphysis where the original tibia diaphysis was not enveloped within the pathologic outgrowth, and it is here that the transverse thin section was obtained (Fig. 4e). The tibia thin section reveals a “core” of non-pathologic diaphyseal primary bone, mostly enveloped in pathologic woven tissue with radial vascular canals. Neither the periosteal margin of the pathological region nor that of the normal bone surface appears mechanically weathered. In transverse section the tibia diaphysis looks as if it were sheared in half, with the posterolateral portion shifted anteriorly as one piece (Fig. 5a). The primary bone of the innermost cortex is fibro-lamellar or poorly organized parallel-fibred, and is poorly organized parallel-fibred within the mid-cortex (Fig. 5b). Throughout the cortex, vascularity varies between simple primary longitudinal canals, anastomosing canals, longitudinal primary osteons, reticular primary osteons, and radial primary osteons. Reticular and radial vascularity is most common on the medial side of the cortex. Four LAGs are visible (Fig. 5c,d). The fourth LAG is very close to the periosteal surface and is only fully visible on the lateral side (Fig. 5d), as it appears to merge with the periosteal surface when followed lateral to medial.

Within the medullary cavity on the anterior side there is extensive radial bone growth from the endosteal surface into the cavity (Fig. 5e). Less pronounced radial endosteal growth is also present on the posterior side, but there is no obvious endosteal boundary separating the new growth from the innermost cortex. Instead, the bone near the endosteal surface is compact coarse cancellous bone that grades into the new endosteal growth. The pathologic tissue found within the medullary cavity joins with the pathologic woven tissue at the periosteal surface both anteriorly and posteriorly, permitted by the gap between the two halves of the tibia (Fig. 5f). The pathologic tissue does not transition smoothly from the primary tissue of the original tibia outer cortex. Instead there is a distinct boundary between “normal” tibia bone tissue and the woven pathologic tissue at what would be considered the periosteal surface of the tibia if it was an unaffected diaphysis. The woven pathologic outgrowth is highly vascularized with radial canals in the posterolateral area (Fig. 5g), but in the posteromedial and anterior regions the vascularity is less strongly radial and more haphazard. Within the woven pathologic tissue of the posterolateral side there are three “bands” of less vascularized tissue (Fig. 5g) formed by a decrease in vascular canal diameter, with longitudinal rather than radial vascularity, very similar in appearance to the cortical outgrowths described in *Maiasaura* tibiae^30^ (Fig. 5h).

### Ontogenetic Trends

**Figure 6:**
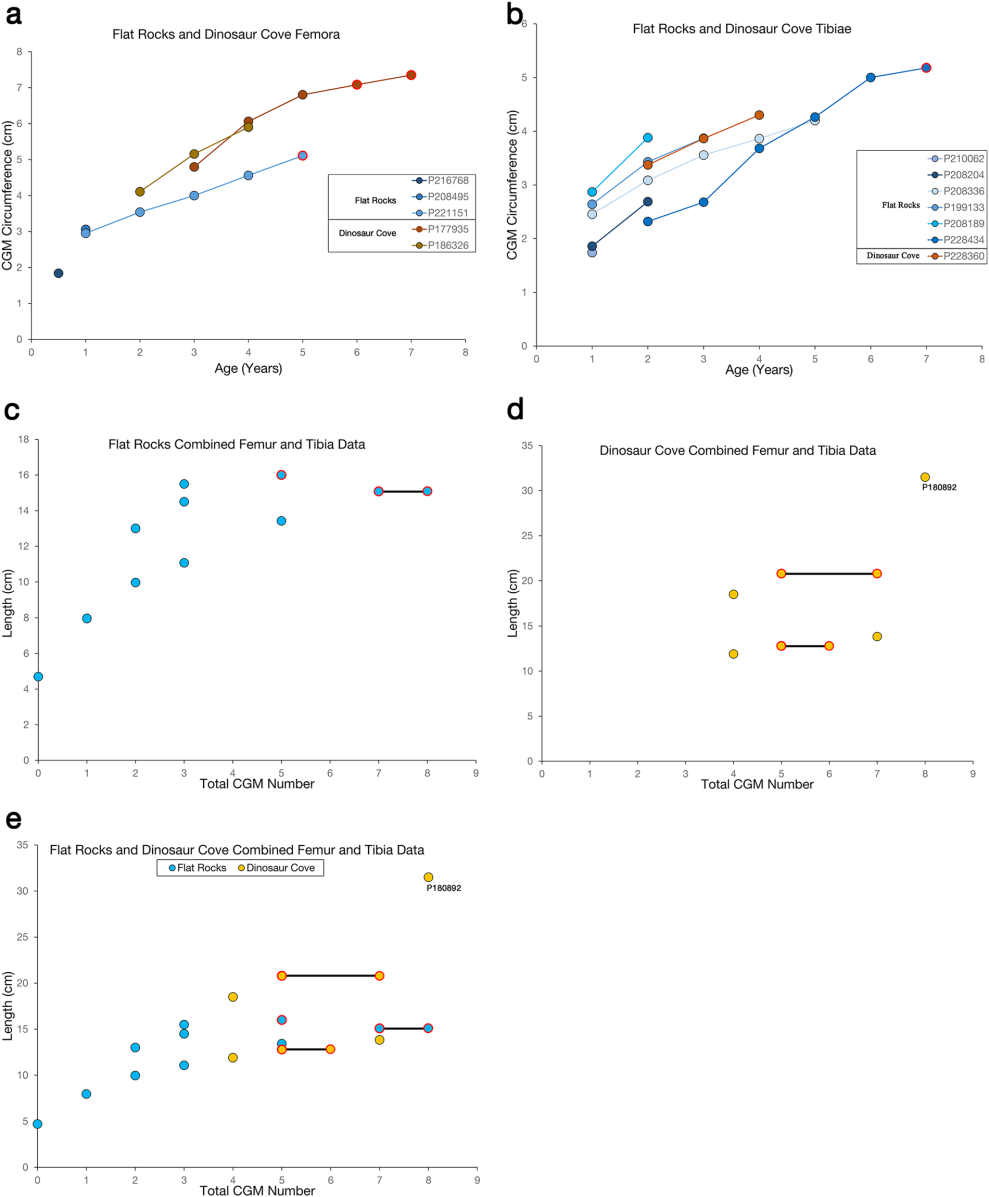
Ontogenetic trends in Victoria hypsilophodontid femora and tibiae. Flat Rocks specimens are plotted in shades of blue, and Dinosaur Cove specimens are plotted in shades of brown. CGMs found within the EFS are outlined in red. (**a**) For Flat Rocks and Dinosaur Cove femora in which a longitudinal growth record was quantifiable, individual age (as assessed by CGM count) is plotted against annual CGM circumference. (**b**) For Flat Rocks and Dinosaur Cove tibiae in which a longitudinal growth record was quantifiable, individual age (as assessed by CGM count) is plotted against annual CGM circumference. (**c**) The total CGM count in each Flat Rocks femur was plotted against total femur length. Tibia lengths for Flat Rocks specimens were converted to corresponding femur lengths and included on the same graph. NMV P208495 and NMV P208189 femur values directly overlap (2 CGMs, femur lengths of 13 cm). A weakly asymptotic trend emerges, with skeletally mature individuals as the oldest and largest specimens. The red bar connects the first and last CGM number within the EFS of a single specimen. (**d**) The total CGM count in each Dinosaur Cove femur was plotted against total femur length. Tibia lengths for Dinosaur Cove specimens were converted to corresponding femur lengths and included on the same graph. No discernible trend emerges. NMV P180892 is likely an outlier. The red bars connect the first and last CGM number within the EFS of a single specimen. (**e**) When the Flat Rocks and Dinosaur Cove femur datasets are plotted together, the Dinosaur Cove specimens (excluding outlier NMV P180892) plot within the range of Flat Rocks data points, contributing to the weakly asymptotic trend. The red bars connect the first and last CGM number within the EFS of a single specimen.

Regarding quantitative analyses, four of the Flat Rocks femora (NMV P216768, NMV P208495, NMV P199058, NMV P221151) are intact enough to allow the periosteal surface, or both the periosteal surface and CGMs, to be fully traced and circumferences quantified, but only femur NMV P221151 has an extensively traceable CGM record (Fig. 6a). On the other hand, every tibia within the Flat Rocks sample possesses traceable CGMs and periosteal surfaces, allowing records of longitudinal femur circumference increase (hereafter referred to as increase in “size”) for multiple specimens (Fig. 6b). The ontogenetically younger tibiae exhibit annual individual variability in size between years one and five, but size trajectories are similar. NMV P228434 is the largest tibia and has the longest ontogenetic record, exhibiting a femur size trajectory similar to the other tibiae between one and four years, but also attaining an asymptote beginning in the seventh year (Fig. 6b). When Flat Rocks tibia lengths are converted to femur lengths and all specimens are plotted together, a weakly asymptotic curve emerges but with considerable scatter (Fig. 6c). The oldest and largest two specimens make up the asymptotic portion of the curve and also possess EFS.

Of the six Dinosaur Cove individuals, two femora (NMV P177935, NMV P186326) have traceable CGMs and periosteal surfaces. The size trajectories of those femora are very similar. Both curves appear to represent the subadult portion, where annual femur circumference increase is reduced approaching skeletal maturity (Fig. 6a). In the case of NMV P177935, the curve becomes asymptotic in the final three years of life. There is only a single Dinosaur Cove tibia (NMV P186334) with longitudinal data. When plotted, the curve is almost linear. Two of the specimens (NMV P177935, NMV P150054) possess an EFS, but the combined Dinosaur Cove dataset, with tibia measurements converted to femur lengths, is not informative (Fig. 6d); no asymptotic trend emerges.

When Flat Rocks and Dinosaur Cove femur datasets are compared, both of the Dinosaur Cove specimens are annually larger than the Flat Rocks ones (Fig. 6a). The combined tibia dataset (Fig. 6b) shows the single Dinosaur Cove specimen with a longitudinal record (NMV P228360) falls within the size trajectories of the Flat Rocks samples, and for two years overlaps the CGM circumferences of Flat Rocks specimen NMV P199133. The Flat Rocks and Dinosaur Cove combined dataset containing tibia lengths converted to femur lengths (Fig. 6e) reveals that the Dinosaur Cove sample falls within the femur length range of the larger Flat Rocks sample and contributes to the weakly asymptotic trend. Skeletal maturity between individuals spans five to seven years. There is a single Dinosaur Cove outlier, NMV P180892, which is much larger than the other samples and which does not have an EFS. And although Dinosaur Cove tibia NMV P228360 falls within the overall trend in size for LAG count, it is one of the ontogenetically oldest specimens and yet lacks an EFS.

## Discussion

In a comparative study on four lower latitude (i.e., non-polar) hypsilophodontid genera by Horner *et al*.^10^, the authors proposed that in some cases a hypsilophodontid genus could, in fact, represent the juvenile form of a different, and larger genus. They suggested this in part because no *Dryosaurus* specimens within their sample were skeletally mature. While this may be the case for *Dryosaurus*, the Australian hypsilophodontids sampled here (excluding NMV P180892) appear to truly be diminutive. This ontogenetic, longitudinal histological assessment reveals that polar hypsilophodontid specimens from the Flat Rocks and Dinosaur Cove localities of Victoria, Australia, were growing like typical small-bodied vertebrates, which tend to have lower growth rates than vertebrates with larger asymptotic body lengths (see Supplementary Table S1 for a summary of growth rates for bone tissue types)^31,32^. For comparison, on average the hadrosaur *Maiasaura* took eight years to reach skeletal maturity with a tibia length of 90 cm^16^, whereas one polar hypsilophodontid (NMV P228434) examined here took seven years to reach skeletal maturity and had a tibia length of 21.8 cm.

The Victoria hypsilophodontids had a relatively elevated growth rate for the first few years, as evidenced by the presence of fibro-lamellar, incipient fibro-lamellar, or poorly organized parallel-fibred tissue prior to the third CGM. Incipient fibro-lamellar tissue resembles rapidly forming fibro-lamellar tissue except that vascular canals are simple or primary osteons are small and less well-developed than primary osteons within truly fibro-lamellar tissue^17,27,28^. Incipient fibro-lamellar tissue is also not sustained throughout the majority of ontogenetic growth in species that form it^17,27,28^. After the second or third CGM, tissue is primarily parallel-fibred, indicating a shift to a decreased growth rate until skeletal maturity was attained. There was no evidence at any ontogenetic stage of extensive cortical remodelling in the form of compact bone converted to cancellous trabeculae, or intensive cortical remodelling by secondary osteons, even after skeletal maturity was attained. This may be a result of small body size and slower growth rates when compared to larger non-avian dinosaurs that exhibit extensive cortical remodelling throughout ontogeny^16,33^.

The innermost cortex of every femur in the sample, as well as two tibiae (NMV P210062 and NMV P228360), possesses regions of compact coarse cancellous bone (CCCB). The location of CCCB around the medullary cavity varies in femora, but is most frequently found on the medial or posteromedial side. It is present on the lateral side of both tibiae. In growing long bones, the metaphyseal regions consist of spongey trabecular bone. As the bone shaft elongates during ontogeny, the spongey metaphyseal regions become incorporated into the diaphysis and the trabeculae are subsequently in-filled with additional primary bone to form compact coarse cancellous bone^21^. Later, medullary drift would erode CCCB on the sides of the medullary cavity being resorbed. Areas of the innermost cortex of hypsilophodontid bones that were not in the process of being resorbed prior to death are lined with a secondarily formed lamellar endosteal layer of varying thickness.

In one tibia (NMV P186334) and every femur with a lamellar endosteal layer, abundant radial channels are embedded in lamellar endosteal tissue, connecting the medullary cavity with primary bone or compact coarse cancellous bone (e.g., Fig. 2e and Supplementary Figures). It is possible these structures are radiating microcracks, which could have formed during the life of the individuals or during fossilization. Alternatively, they could have housed vascular canals, but the endosteal layer would have grown around them as there is no indication of resorption cavities through the endosteal layer to form them. A similar morphology was reported and figured in diaphyses of both Mesozoic and extant avian bone^34–37^, supporting the latter hypothesis. Comparative examination of diaphyseal thin sections used in the lower latitude hypsilophodontid study by Horner *et al*.^10^ revealed intact, avascular endosteal lamellar bone, free of radial canals. Radial vascular canals within the lamellar endosteal layer of the high-latitude Victoria hypsilophodontids therefore marks a histological difference between them, and the lower latitude specimens utilized in that earlier study, requiring further exploration into the biologic or mechanical origin of those canals.

Regarding the graphs of CGM counts plotted against CGM circumference and element length, there is not strong evidence for separate genera amongst or within the Flat Rocks or Dinosaur Cove samples, except for NMV P180892 (Fig. 6d-e). That specimen is much larger than the other femora in either dataset, and was initially included to test the hypothesis that the majority of Victoria hypsilophodontid specimens represented ontogenetically immature individuals of a species that grew much larger. Because NMV P180892 has at least eight CGMs and there is no indication of an EFS, it is very likely that this fragmentary femur represents a separate genus. In all other cases, it is difficult at present to differentiate between annual individual variation in femur size within a single taxon and differences due to a sample consisting of multiple genera. Dinosaur Cove tibia NMV P228360 closely follows the size trajectory of Flat Rocks tibia NMV P199133 for the few CGMs that could be quantified, but this skeletally immature individual is one of the oldest in the sample (7 CGMs preserved). This, despite being very similar in size to skeletally mature Flat Rocks tibia NMV P228434 (Fig. 6e). While an argument could be made that the trajectories of Flat Rocks tibiae NMV P210062 and NMV P208189 are much steeper than the other tibiae and therefore represent accelerated growth rates and potentially different genera, such an argument would be based on 2–3 data points forming incomplete curves. Large-sample histological analyses demonstrate considerable individual variation in size over ontogeny in some dinosaur genera^16,38^. Results of such studies caution against proposing the presence of multiple genera here until the dataset can be augmented with additional specimens.

The reassessment of thin section slides produced by Chinsamy *et al*.^4^ (labelled AI-AIII) from NMV P177935 (Fig. 2) confirm no CGMs are visible, but the explanation for this absence is technical rather than biological. In an intraskeletal alligator histology study, Woodward *et al*.^17^ remark on slides that they polished too thin, observing that CGMs within alligator diaphyses which were clearly visible on one slide, were difficult to recognize or non-existent on duplicate slides of the same specimen when polished too thin. It appears that the same situation occurred with slides AI and AII, providing an explanation for why no CGMs were observed in this femur in Chinsamy *et al*.^4^.

Concerning the femur and tibia of NMV P186047, the femur reveals none of the pathologic symptoms of the contralateral tibia in either hand sample or in thin section (Fig. 4). Gross *et al*.^12^ state that based on x-rays, the tibia ceased growth for a while and then resumed, and the animal lived with the infection for some time. The histology of the non-pathologic femur tends to confirm this observation: within the cortex there are two LAGs within a thick annulus, and this CGM combination was not observed in any other hypsilophodontid femur examined (Fig. 4c). An annulus represents a period of decreased growth rate, while a LAG represents a growth hiatus. Despite the close proximity of the two LAGs, they do not overlap or merge around the cortex and so are interpreted to represent the growth hiatus of two separate years. The close CGM spacing, within an annulus, suggests little growth in the corresponding year. This decreased growth may relate to the onset of infection, but more foundational studies on causes or influences of growth mark formation using extant taxa are necessary before this hypothesis can be properly tested. The femur has a small EFS, and a single LAG within the EFS (Fig. 4d). Assuming no missing CGMs, the individual would have been skeletally mature at five years of age and within its seventh year of life when it died. However, it is unclear how the injury would have affected, or possibly truncated, growth to a larger asymptotic size.

Based on morphology, the pathology displayed by the left tibia was diagnosed as acute osteomyelitis (Fig. 4e)^12^. Gross *et al*.^12^ previously stated that there is no evidence of bone fracture from the outward morphology, and this was confirmed by the senior author prior to sectioning. However, the histological analysis shows that the tibia was indeed fractured. This fracture could, therefore, have been the source of the resulting infection. In addition, a lateral tissue outgrowth with radial vascularity was observed in the tibia of NMV P186047, and its histologic appearance closely resembles the outgrowths described in tibiae of *Maiasaura*^30^, including thickened bands of tissue resulting from reduced vascularity. The outgrowths in *Maiasaura* were hypothesized to result from the tibia compensating for additional load bearing after fibular failure, with the thickened banding of decreased vascularity occurring to strengthen the additional bone for weight support. Although the left fibula is incomplete in NMV 186047, if it too were fractured at the same time as the tibia, the response may have been compensation for the fractured fibula through deposition of new radial woven tissue on the lateral side of the tibia. Three LAGs are visible within the mid-cortex of the non-pathologic portion of the bone. A fourth LAG is so close to what was the “normal” periosteal surface that it in some places merges with it. Since the pathologic growth is present after the fourth LAG, it is reasonable to suggest that the injury resulting in the outgrowth occurred after the fourth year, assuming no LAGs are missing due to medullary cavity enlargement.

This study is the first multi-specimen ontogenetic histological analysis of Australian polar hypsilophodontids. It demonstrates that sixteen of the seventeen individuals examined were asymptotically small-bodied dinosaurs and that skeletal maturity was attained in five to seven years. Because of its considerably larger size, minimum 8 LAGs, and no EFS, NVM P180892 is most likely a separate genus. Otherwise there is no appreciable difference in size trajectories within or between Flat Rocks and Dinosaur Cove samples. Based on the available histological data, a single genus of small-bodied hypsilophodontid is represented within the Victoria sample examined thus far. However, a single genus seems unlikely considering the geologic interval between the Flat Rocks and Dinosaur Cove localities (approximately 26 Ma) and the distribution of EFS timing. This study therefore demonstrates the potential limitations associated with small sample sizes when attempting detailed taxonomic and ontogenetic histology-based interpretations, and the importance of utilizing multiple lines of evidence (e.g., geologic interval, morphology, etc.) when assessing life histories of extinct taxa. Regardless, results of this study should not dissuade histological research using limited data, as important life history insights at the individual level are still obtainable and critical for foundational knowledge. Instead, the results of this study encourage cautious interpretations of life history parameters from such datasets, and acknowledgement of their inherent limitations. Ultimately, more histologic sampling of Victoria hypsilophodontids is necessary to go beyond the conservative, parsimonious explanation of individual variation in size in order to assess the possibility of multiple genera existing within or between the Flat Rocks and Dinosaur Cove ecosystems represented by the specimens collected from Victoria. In particular, more specimens surviving to skeletal maturity are necessary to properly assess individual variation in size trajectories throughout ontogeny.

## Methods

Isolated, often fragmentary postcranial hypsilophodontid elements are common in the Early Cretaceous sediments of Victoria, but due to conservative postcranial morphology, these specimens are difficult to taxonomically assign beyond “hypsilophodontid”^7^. In a previous study, femora and tibiae of unassigned hypsilophodontids from the Museum Victoria (NMV, Melbourne, Australia) were histologically sectioned and digitally imaged to assess the presence of annually deposited cyclical growth marks (CGMs) and to examine bone tissue organization (see Woodward *et al*.^6^ for a description of thin section processing and image acquisition methodology). Here, the thin sections and images produced for Woodward *et al*.^6^ are used to assess ontogenetic age, longitudinal femur circumference trajectories, and provide detailed bone tissue microstructure descriptions. Additional photomicrographs of the thin section slides prepared for Woodward *et al*.^6^ were obtained for this study using a Nikon Ni-U microscope fitted with a Nikon Ri-2 camera and an ASI MS-2000 motorized stage.

Samples from the Valanginian-Barremian Flat Rocks locality consist of five femora and six tibiae. The Dinosaur Cove locality (Albian in age) sample incorporates four femora and three tibiae, including the right femur and left tibia from the articulated posterior skeleton of NMV P186047. The left tibia of NMV P186047 is pathologic and its morphology was previously described by Gross *et al*.^12^ and diagnosed as a case of acute osteomyelitis. Three specimens from the Dinosaur Cove locality (NMV P150054, NMV P177935, and NMV P186326) are assigned to *Fulgurotherium australe*. The remainder of the histological sample described here is not taxonomically assigned beyond the generalization “hypsilophodontid”.

The present study also includes a thin section prepared for Woodward *et al*.^6^ from the same femur (NMV P177935) described by Chinsamy *et al*.^4^ as lacking CGMs. Using the new thin section slide, Woodward *et al*.^6^ demonstrated the presence of CGMs within this specimen but did not include a detailed histologic description. The original thin section slides of NMV P177935 produced for Chinsamy *et al*.^4^ as well as the thin section slide produced by Woodward *et al*.^6^ are also described here. Detailed histological descriptions and figures for each hypsilophodontid specimen listed in Table 1 not described within the results section is provided in Supplementary Information online. For all qualitative descriptions, bone microstructure terminology follows that set forth by Francillon-Vieillot *et al*.^39^ unless otherwise noted.

The length of each complete femur was measured as a straight line distance from the top of the femoral head to the bottom of the lateral condyle. Tibia length was measured from the top of the cnemial crest to the bottom of the lateral condyle. For partial femora and tibiae, images were superimposed onto photos of complete elements as additional layers in Adobe Photoshop CC. The images of complete elements were then scaled up or down until they matched the proportions of the partial elements. The measurement tool in Adobe Photoshop was then used to obtain a length estimate for the incomplete elements (Table 1). For all transverse thin sections, medullary cavity and periosteal surface circumferences were traced in Adobe Photoshop CC. Cyclical growth marks (CGMs), when present, were also digitally traced. CGMs include both lines of arrested growth (LAGs) and annuli. A LAG is a hypermineralised line within the cortex representing a brief cessation of appositional bone growth, while an annulus is a band of often well-organized and less vascularized tissue representing a brief period of decreased appositional growth^39^. Both CGMs resemble tree rings in transverse thin section and form annually in extant tetrapods for which data exist^40–42^. By extension, CGMs present in fossil bone are assumed to have formed annually and so can be used to obtain an ontogenetic age. Within the sample set, in some instances extensive taphonomic crushing made fully tracing CGMs impossible. In other cases, due to medullary cavity expansion, the innermost CGMs were partially obliterated. However, if enough of the partial CGM was still present, a reasonable estimation of the completed CGM circumference was attempted, but this was not always possible. Medullary, CGM, and periosteal surface circumferences for each specimen were quantified using ImageJ^43^ software and the BoneJ^44^ plugin. Medullary cavity area, cortical bone area, and zone area were also determined using the BoneJ plugin. Major and minor axis diameters were determined within Adobe Photoshop CC using the measurement tool (Supplementary Table S2).

To account for the possibility of missing CGMs due to medullary cavity expansion, retrocalculation^18–20^ was performed using the digitally traced CGM circumferences of the smaller (and presumably ontogenetically younger) tibiae and femora. In addition to their small size with small medullary cavity diameters, these specimens had fibro-lamellar to poorly organized parallel-fibred tissue and between zero and two CGMs, suggesting these were immature individuals and providing further support that these elements were not themselves missing any CGMs.

Using Adobe Photoshop CC, the CGM circumferences of the smallest elements were digitally transposed onto the medullary cavity of larger elements in an attempt to match the circumferences of partial CGMs within the latter and thereby confirm that the innermost partial CGM was indeed the first CGM (i.e., formed at the end of the first year), or to estimate the number of CGMs potentially missing due to medullary cavity enlargement.

Due to the small sample size resulting in few data points (i.e., mixed elements consisting of eleven independent Flat Rocks specimens, and five independent Dinosaur Cove specimens), construction of body mass curves was not attempted. Plots of CGM counts versus element length were made instead (Fig. 6). In addition, for elements with traceable CGMs, longitudinal (i.e., ontogenetic) plots were made of CGM number versus yearly CGM circumference (Fig. 6). However, unless an individual perished during the annual growth hiatus, the periosteal surface circumference value does not represent the circumference of a true CGM. With isolated skeletal elements, it is difficult to determine whether or not the individual died during the growth hiatus^17^. Therefore, if the specimen did not have an EFS, the circumference of the periosteal surface was omitted from the growth trajectory plot of the immature individual.

Again because of a small sample set, and because the sample consists of both femora and tibiae, meaningful ontogenetic comparisons within and across the Flat Rocks and Dinosaur Cove datasets is limited. To circumvent this problem, tibia lengths in each sample were converted to corresponding femur lengths based on the femur:tibia length ratio (0.69) calculated for an articulated hypsilophodontid hindlimb in the Museum Victoria collections (NMV P186047, non-pathologic right hindlimb) and assuming ontogenetic isometry. Although such a procedure introduces error, it enables a visual comparison of CGM count versus element length across all specimens and so affords tentative observations (Fig. 6).

### Data Availability

Hypsilophodontid fossils and resulting thin section slides produced for Woodward *et al*.^6^ utilized here are reposited at Museums Victoria, Victoria, Australia. Images of all thin section specimens described herein and in the supplementary files are available online at Morphobank.org (http://morphobank.org/permalink/?P2625), and higher resolution images are provided by the senior author upon request.

## Electronic supplementary material

Supplementary Files
