# Supplementary material for: The bone microstructure of polar “hypsilophodontid” dinosaurs from Victoria, Australia
Source: Sci Rep. 2018 Jan 18;8:1162. doi: 10.1038/s41598-018-19362-6 (PMC5773672; doi:10.1038/s41598-018-19362-6)
Supplement: Supplementary file 1 — Supplementary Files [file 41598_2018_19362_MOESM1_ESM.pdf]

The bone microstructure of polar “hypsilophodontid” dinosaurs from Victoria, Australia

Holly N. Woodward[1*], Thomas H. Rich[2,3], Patricia Vickers-Rich[2,3,4,5]

[*]Email: holly.n.woodward@gmail.com
[1]Oklahoma State University Center for Health Sciences, Tulsa, Oklahoma, United States of America
[2]Museums Victoria, Melbourne, Victoria, Australia
[3]Swinburne University of Science and Technology, Melbourne, Victoria, Australia
[4]School of Earth, Atmosphere and Environment, Monash University, Melbourne, Victoria, Australia
[5]Deakin University, Melbourne, Victoria, Australia

Supplementary Text

Detailed histological descriptions of hypsilophodontid femora and tibiae from the collections of Museum Victoria (NMV), Victoria, Australia.

Flat Rocks Locality:
<u>NMV P208495</u> (left femur, Supplementary Figure 1): Compact coarse cancellous tissue is located on the posteromedial side and separated from the medullary cavity by a lamellar endosteal layer. This lamellar endosteal layer is only present on the medial side of the femur. Radial channels pierce through the lamellar endosteal layer. Primary tissue throughout the cortex varies between poorly organized parallel-fibred to parallel-fibred, with small longitudinal primary osteons. Osteocyte lacunae are dense and plump within the cortex but are flattened in the outermost cortex near the periosteal surface. Two LAGs are visible. The first (innermost) LAG is partially destroyed by medullary expansion, while the second LAG is within the outermost cortex. Because the periosteal surface is mechanically eroded in places, the perimeter of the second LAG is not fully traceable.

<u>NMV P199058</u> (left femur, Supplementary Figure 2): Preservation of bone microstructure is poor, possibly due to microbial invasion. On the posteromedial side adjacent to the medullary cavity this is an area of compact coarse cancellous tissue. A lamellar endosteal layer surrounds the medullary cavity and is thickest on the posteromedial side. The endosteal layers is pierced by numerous radial channels. There are three LAGs. Prior to the first LAG, and for a short distance after it, tissue is poorly organized parallel-fibred. Osteocyte lacunae in this area are dense and plump. Between the first and second LAG the tissue becomes mostly parallel-fibred and osteocyte lacunae are flattened. Vascular canal orientation is primarily longitudinal but more laminar in places. The innermost LAG, which is followed by an annulus, is truncated on the lateral side due to medullary expansion.

<u>NMV P221151</u> (left femur, Supplementary Figure 3): Tissue preservation is poor and there is scattered pyrite invasion. Compact coarse cancellous bone is present on the posterior side of the medullary cavity. This area is separated from the medullary cavity by a thin lamellar endosteal layer. Cortical primary tissue is poorly organized parallel-fibred with longitudinal primary osteons. Osteocyte lacunae within the inner and mid-cortex are dense and rounded to somewhat flattened. There are five LAGs within the cortex, three of which are truncated by medullary drift. The outermost cortex is lamellar with dense, flattened osteocyte lacunae. Vascularity is sparse and consists of anastomosing simple vascular canals. The transition to primarily lamellar avascular bone at the periosteal surface after the fifth LAG may indicate the beginnings of an EFS, but it is thin.

<u>NMV P180892</u> (left femur, Supplementary Figure 4): The innermost cortex on all but the medial side consists of compact coarse cancellous bone and scattered secondary osteons. The innermost cortex on the medial side is primary periosteal tissue, which was in the process of being resorbed due to medullary drift. On the lateral side, bone tissue is remodelled to the mid-cortex. The lamellar endosteal layer encircling the medullary cavity is thickest on the anterolateral side. Here, radial channels are present within the thick (1.5 mm) endosteal layer and open into the medullary cavity. Primary tissue within the cortex is poorly organized parallel-fibred with longitudinal simple vascular canals. Seven LAGs are within the cortex, and an eighth is within 50 microns of the periosteal surface. No EFS is visible, suggesting this individual was not skeletally mature even with a femur circumference of 10.08 cm, considerably larger than other samples.

<u>NMV P150054</u> (right femur, Supplementary Figure 5): The cortex is crushed on the lateral side and fragments fill the medullary cavity. A small patch of compact coarse cancellous bone is visible on the medial side. Large secondary osteons are visible on the medial side in two groups, one in the inner cortex, and the other in the outer cortex. Otherwise the cortex is primary bone. The innermost primary cortical

tissue is a combination of fibro-lamellar and poorly organized parallel-fibred, with small or incipient longitudinal primary osteons. Osteocyte lacunae density is high in the inner and mid-cortex but is lower near the periosteal surface. The cortex becomes more strongly parallel-fibred in the outer cortex. Within the cortex are three CGMs. The first two are LAGs, while the third is an annulus.

NMV P210062 (right tibia, Supplementary Figure 6): The primary cortical tissue varies between reticular and longitudinal fibro-lamellar, and poorly organized parallel-fibred. There is an area of compact coarse cancellous bone on the lateral side bordering the medullary cavity. A lamellar endosteal layer is located on the anterior surface bordering the medullary cavity. Secondary osteons are dense and confined to the lateral side from the endosteal to periosteal surface, forming the anterolateral plug of the tibial border[4]. There is a LAG present within the cortex but partially obliterated on the medial side by medullary expansion, and laterally by secondary osteons. Opaque areas of the slide are due to pyrite.

NMV P208204 (right tibia, Supplementary Figure 7): The innermost cortex of the anterior and posterior sides is fibro-lamellar, but otherwise cortex is parallel-fibred. Vascularity is longitudinal to anastomosing simple vascular canals and small primary osteons. Two LAGs are visible within the cortex. Osteocyte lacunae density is high prior to the first LAG but is less dense thereafter.

NMV P208336 (left tibia, Supplementary Figure 8): Preservation of bone microstructure is poor, and the tissue suffers from pyrite invasion. However, the cortex appears parallel-fibred with longitudinal simple primary vascular canals. There is a thin (68 μm) lamellar endosteal layer. Five LAGs are visible, the innermost of which is partially destroyed by the enlarging medullary cavity.

NMV P199133 (right tibia, Supplementary Figure 9): The specimen is crushed and tissue is poorly preserved. Pyrite invasion is prevalent. Cortex is parallel-fibred with mostly longitudinal, but also some reticular, simple primary vascular canals. There is a thin (42 μm) lamellar endosteal layer lining the medullary cavity. Three LAGs are visible within the cortex.

NMV P208189 (right tibia, Supplementary Figure 10): The innermost cortex is fibro-lamellar with longitudinal and reticular primary osteons, and there is no lamellar endosteal layer. Elsewhere tissue is parallel-fibred, with either small primary osteons or simple vascular canals in reticular and anastomosing arrangements. Secondary osteons are present from the endosteal to periosteal surface on the anterolateral side, within the region of the anterolateral plug. Two LAGS are within the cortex. Vascular canals and cracks are frequently invaded by pyrite.

NMV P228434 (left tibia, Supplementary Figure 11): The cortex is crushed, with poor tissue preservation and pyrite invasion in several locations. Seven LAGs are within the parallel-fibred bone matrix. Vascular canals are simple anastomosing and longitudinal. A thin (58 μm) lamellar endosteal layer lines the medullary cavity. Osteocyte lacunae density decreases from inner to outer cortex. The LAGs often appear as tightly stacked clusters of two or three lines, but because the lines merge and split when traced around the circumference, each grouping is considered to represent a single annual event. There is an eighth LAG very close to the periosteal surface in a region of decreased vascularity, suggesting a thin EFS is present.

Dinosaur Cove Locality:
NMV P186326 (left femur; Supplementary Figure 12): Compact coarse cancellous bone comprises the innermost cortex on the posteromedial and posterolateral sides. The entire medullary cavity is surrounded by an endosteal layer up to 440 μm thick posteriorly. Frequent radial channels are found through the thick endosteal layer. Bone fibre orientation throughout the transverse section is poorly organized parallel-fibred. Vascularity consists of anastomosing simple canals in the inner and mid-cortex, and is longitudinal in the outer cortex. Osteocyte lacunae density is high. There are three LAGs within the cortex.

NMV P186334 (right tibia; Supplementary Figure 13): The medullary cavity is lined with a lamellar endosteal layer. Radial channels are within the endosteal layer between the primary tissue and the medullary cavity. The innermost primary cortex is fibro-lamellar with small longitudinal primary osteons and anastomosing simple primary vascular canals, and therefore resembles the incipient fibro-lamellar bone[1,5] observed in some modern alligators. Osteocyte lacunae are frequent in the innermost cortex but sparse by mid cortex. From middle to outer cortex, tissue is parallel-fibred, and is lamellar in the outermost cortex. There is a band of colour change in the outermost cortex, but appears to be due to mineral staining rather than a transition to EFS, as tissue organization and degree of vascularity remains the same when examined using polarized light. There are four LAGs within the cortex, the innermost of which is only visible on the anterolateral side due to medullary cavity enlargement. This tibia has a very pronounced anterior-lateral border. The inner to outer cortex of the anterior border is largely secondary.

NMV P228360 (right tibia; Supplementary Figure 14): Compact coarse cancellous bone is concentrated on the lateral side of the innermost cortex, and separated from the medullary cavity by a lamellar endosteal layer. The primary tissue of the innermost cortex varies between fibro-lamellar and very poorly organized parallel-fibred tissue, with longitudinal primary osteons. The cortex is parallel-fibred from middle to outer cortex with longitudinal primary osteons and anastomosing simple vascular canals. Osteocyte lacunae density is high, and lacunae become more flattened in the mid and outer cortex. There are seven annuli within the cortex. Osteocyte lacunae density is greatly decreased in the zone between the 5th and 6th annulus.

Supplementary References:

1 Klein, N. Long bone histology of Sauropterygia from the Lower Muschelkalk of the Germanic Basin provides unexpected implications for phylogeny. *Plos One* **5**, e11613, doi:10.1371/journal.pone.0011613 (2010).

2 Woodward, H. N., Horner, J. R. & Farlow, J. O. Quantification of intraskeletal histovariability in *Alligator mississippiensis* and implications for vertebrate osteohistology. *PeerJ* **2**, e422, doi:10.7717/peerj.422 (2014).

3 Hübner, T. R. Bone histology in *Dysalotosaurus lettowvorbecki* (Ornithischia: Iguanodontia) - variation, growth, and implications. *Plos One* **7**, e22958, doi:10.1371/journal.pone.0029958 (2012).

4 Konietzkoo-Meier, D. & Klein, N. Unique growth pattern of Metoposaurus diagnosticus krasiejowensis (Amphibia, Temnospondyli) from the Upper Triassic of Krasiejów, Poland. *Palaeogeography, Palaeoclimatology, Palaeoecology* **370**, 145-157 (2013).

Supplementary Figures:

Figure S1.

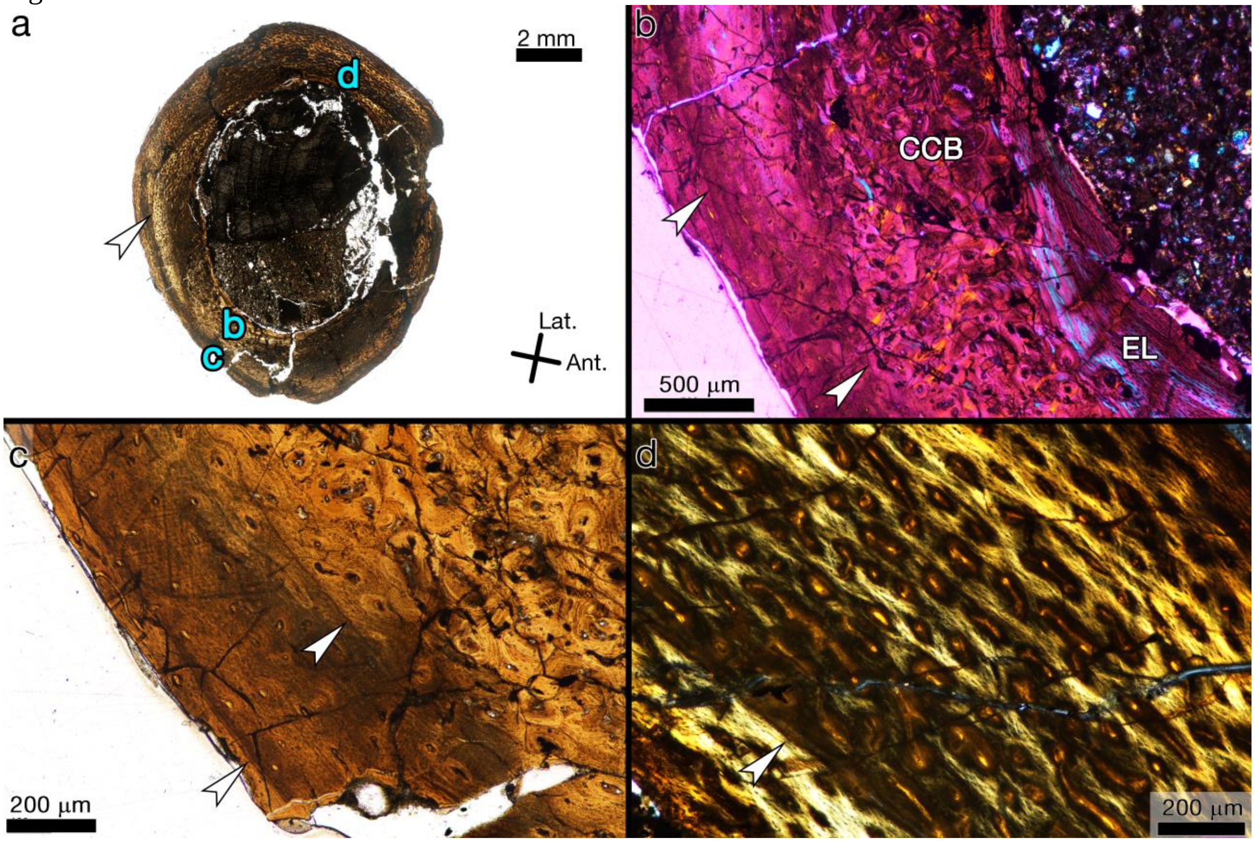

Supplementary Figure 1. Transverse section of left ornithopod femur NMV P208495. a) Image of complete thin section. Blue letters reference the magnified regions shown in corresponding panels. Asymmetrical cortical growth becomes evident when the second LAG (arrow) is traced around the cortex. There is carbonized plant material within the medullary cavity in addition to mineral infilling. Plane polarized light. b) A thick lamellar endosteal layer separates compact coarse cancellous tissue from the medullary cavity on the posteromedial side. Two LAGs are visible within the cortex (arrows). The first (innermost) is partially destroyed by medullary cavity expansion while the second LAG is not fully traceable due to mechanical erosion of the bone. CCB = compact coarse cancellous bone, EL = lamellar endosteal layer. Full wave plate. c) The LAGs (arrows) in panel b are somewhat more visible in plane polarized light. d) Small longitudinal primary osteons are embedded within parallel-fibred tissue on the lateral side of the cortex. The second, asymmetrical, LAG (arrow) is still visible but the first LAG has been destroyed by medullary expansion in this area. Circularly polarized light.

Figure S2.

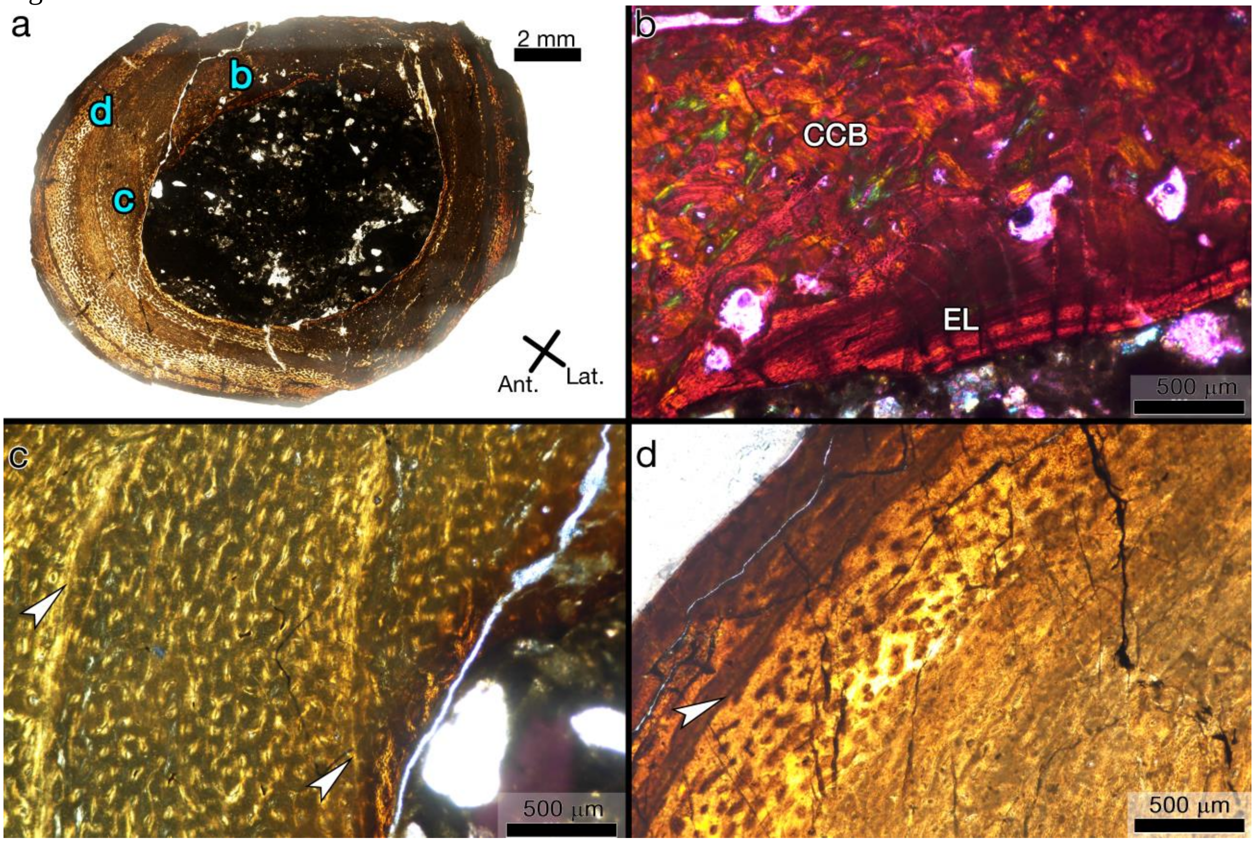

<u>Supplementary Figure 2.</u> Transverse section of left ornithopod femur NMV P199058. a) Image of complete thin section. Preservation quality is poor. Blue letters reference the magnified regions shown in corresponding panels. Plane polarized light. b) A lamellar endosteal layer surrounds the medullary cavity and is thickest on the medial side, where it lines a region of compact coarse cancellous tissue. This endosteal layer is pierced by many radial channels. CCB = compact coarse cancellous bone; EL = lamellar endosteal layer. Full wave plate. c) Two LAGs (arrows) are visible within an inner and mid-cortex of poorly organized parallel-fibred tissue. Viewed in circularly polarized light, the cortex immediately adjacent to each LAG is more parallel-fibred than the surround tissue. d) A third LAG (arrow) is visible in the outer cortex. Although cortical structure is poorly preserved, vascular canals within the outer cortex appear to be simple longitudinal or reticular. Plane polarized light.

Figure S3.

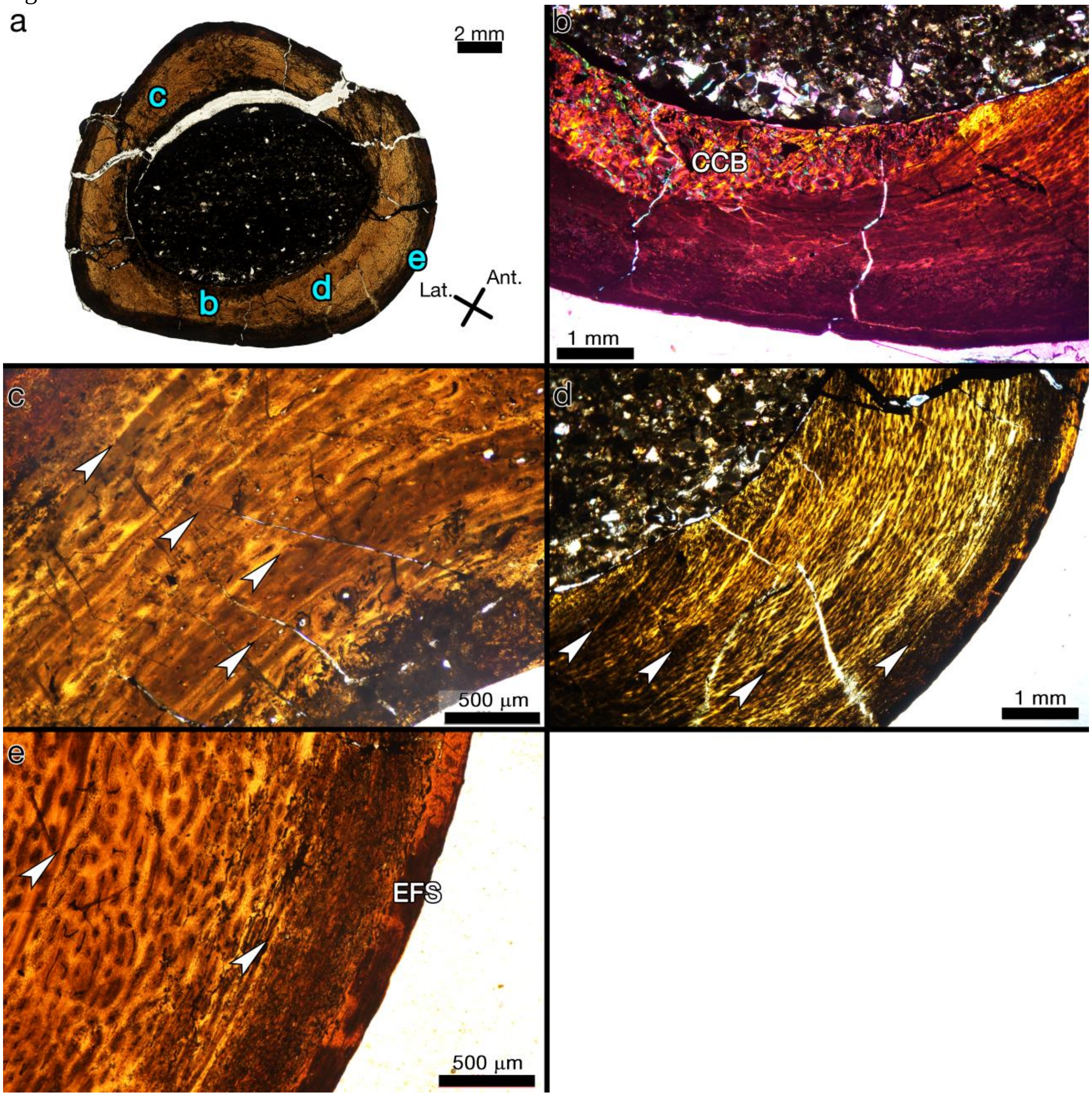

Supplementary Figure 3. Transverse section of left ornithopod femur NMV P221151. Blue letters reference the magnified regions shown in corresponding panels. Plane polarized light. b) A thin lamellar endosteal layer separates the medullary cavity from a region of compact coarse cancellous bone on the posterior side of the inner cortex. CCB = compact coarse cancellous bone. Full wave plate. c) Five LAGs are present in the cortex, and the innermost four (arrows) can be seen in this panel. Plane polarized light. d) The cortex is entirely parallel-fibred with simple vascular canals. LAGs 2-5 (arrows) can be traced in this area. The second and third LAG are partially destroyed by medullary drift. Circularly polarized light. e) The fourth and fifth LAG (arrows) are near the periosteal surface. Although the tissue fibre quality is degraded, fibre orientation appears parallel-fibred to lamellar after the fifth LAG, and may represent a small EFS. EFS = external fundamental system. Plane polarized light.

Figure S4.

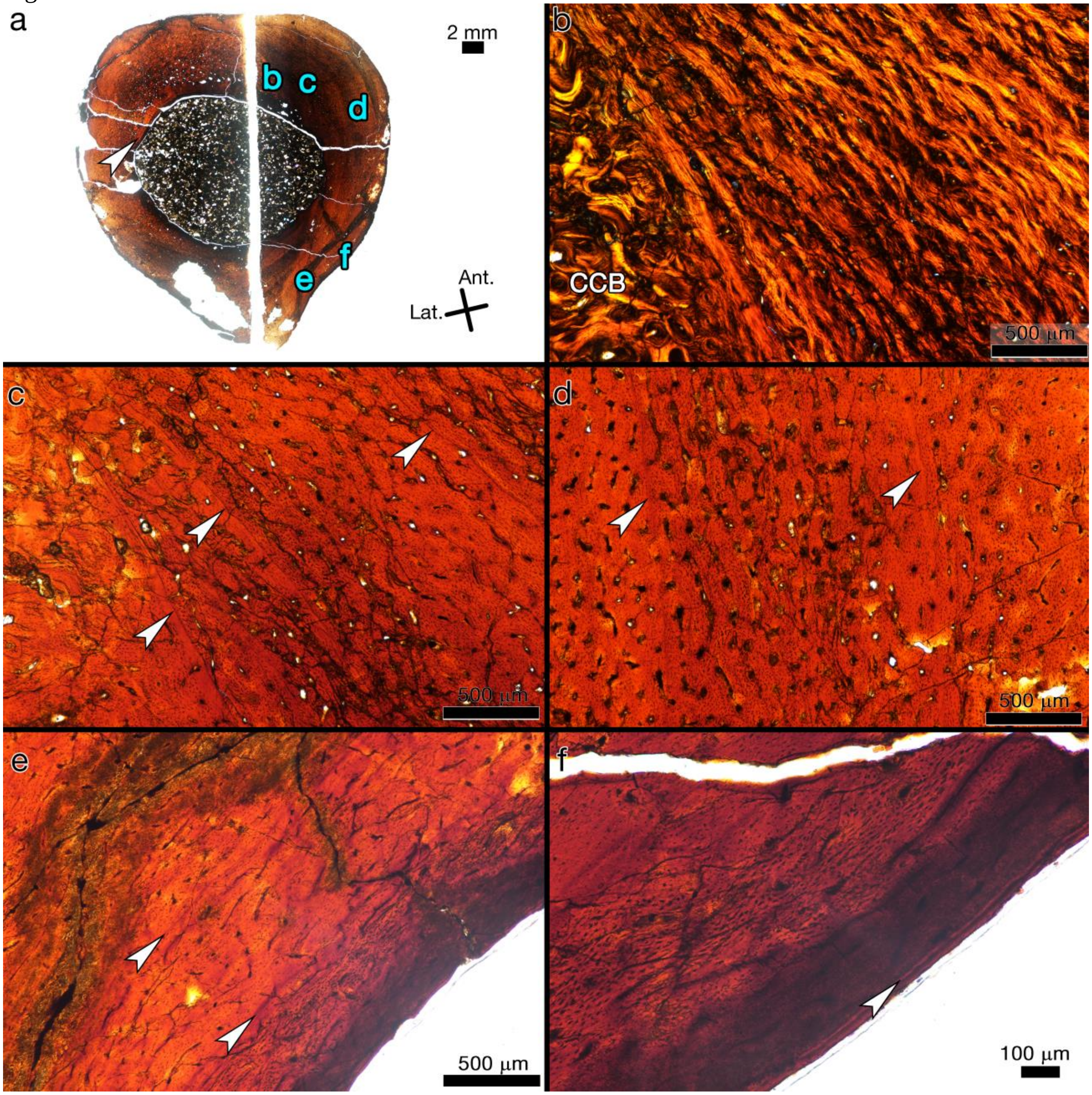

Supplementary Figure 4. Transverse section of left ornithopod femur NMV P180892. a) Composite image of complete thin section. The vertical white line bisecting lateral and medial sides indicates that the femur was cut into two sections, and the medial and lateral halves were mounted to separate slides. The lamellar endosteal layer (arrow) is thickest on the anterolateral side. Resorption of the innermost cortex was occurring on the medial side of the medullary cavity prior to death. Blue letters reference the magnified regions shown in corresponding panels. Quarter wave plate. b) The innermost cortex on all but the medial side consists of compact coarse cancellous tissue, and the primary cortical tissue is poorly organized parallel-fibred with simple longitudinal vascular canals. Circularly polarized light. CCB = compact coarse cancellous bone. c) Three LAGs are visible in the innermost cortex (arrows). The first and second LAGs are not parallel to each other, revealing asymmetrical cortical growth within the intervening year. Plane polarized light. d) Mid-cortex, two more LAGs (arrows) are visible. Plane polarized light. e) A sixth and seventh LAG (arrows) is found within the outermost cortex. f) An eighth LAG (arrow) is visible within 50 microns of the periosteal surface. Plane polarized light.

Figure S5.

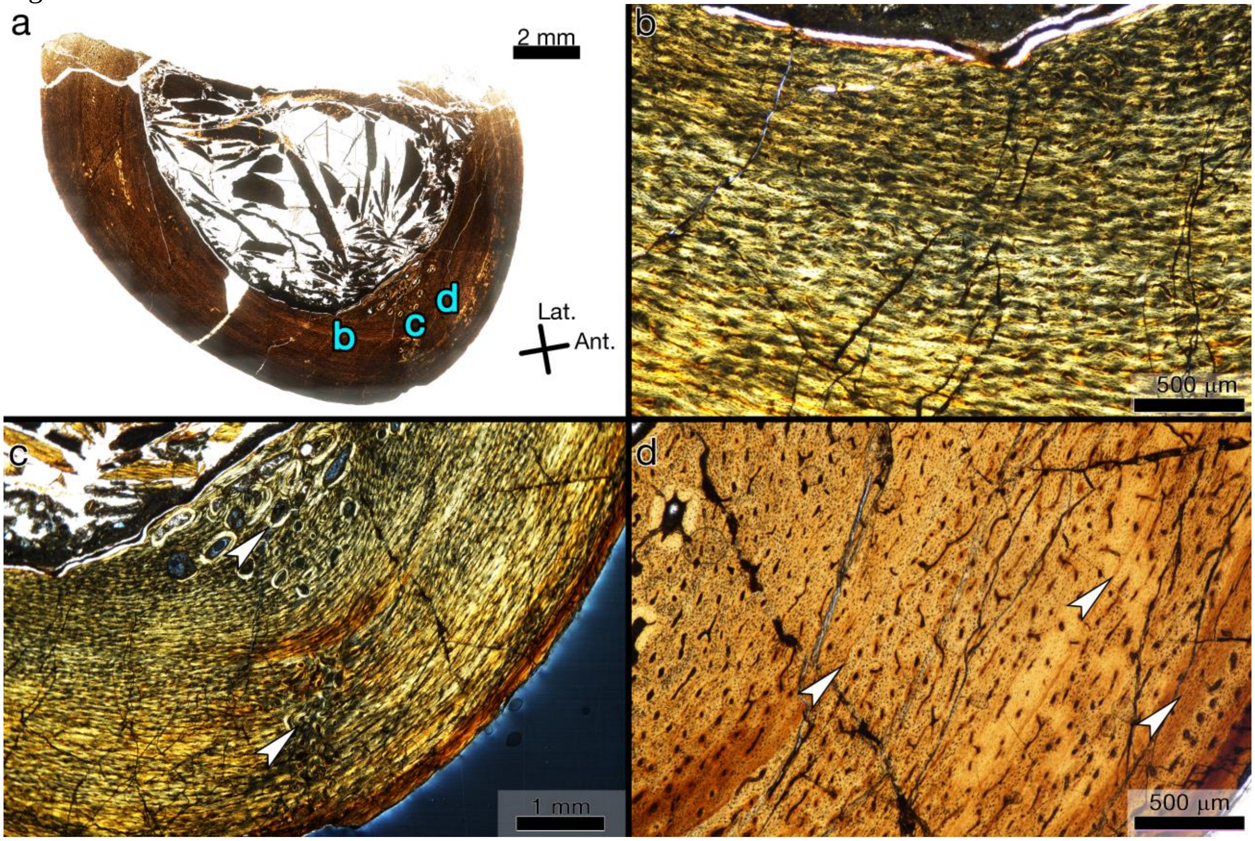

Supplementary Figure 5. Transverse section of right ornithopod femur NMV P150054. Cortex is crushed on the lateral side. Plane polarized light. a) Image of complete thin section. Blue letters reference the magnified regions shown in corresponding panels. Plane polarized light. b) The entire cortex is made of parallel-fibred cortex with longitudinal or anastomosing incipient primary osteons. Circularly polarized light. c) Large secondary osteons cluster in the inner and outer cortex (arrows) within parallel-fibred tissue. Circularly polarized light. d) Three CGMs can be traced within the cortex. The inner two are LAGs, while the outermost is an annulus (arrows). Plane polarized light.

Figure S6.

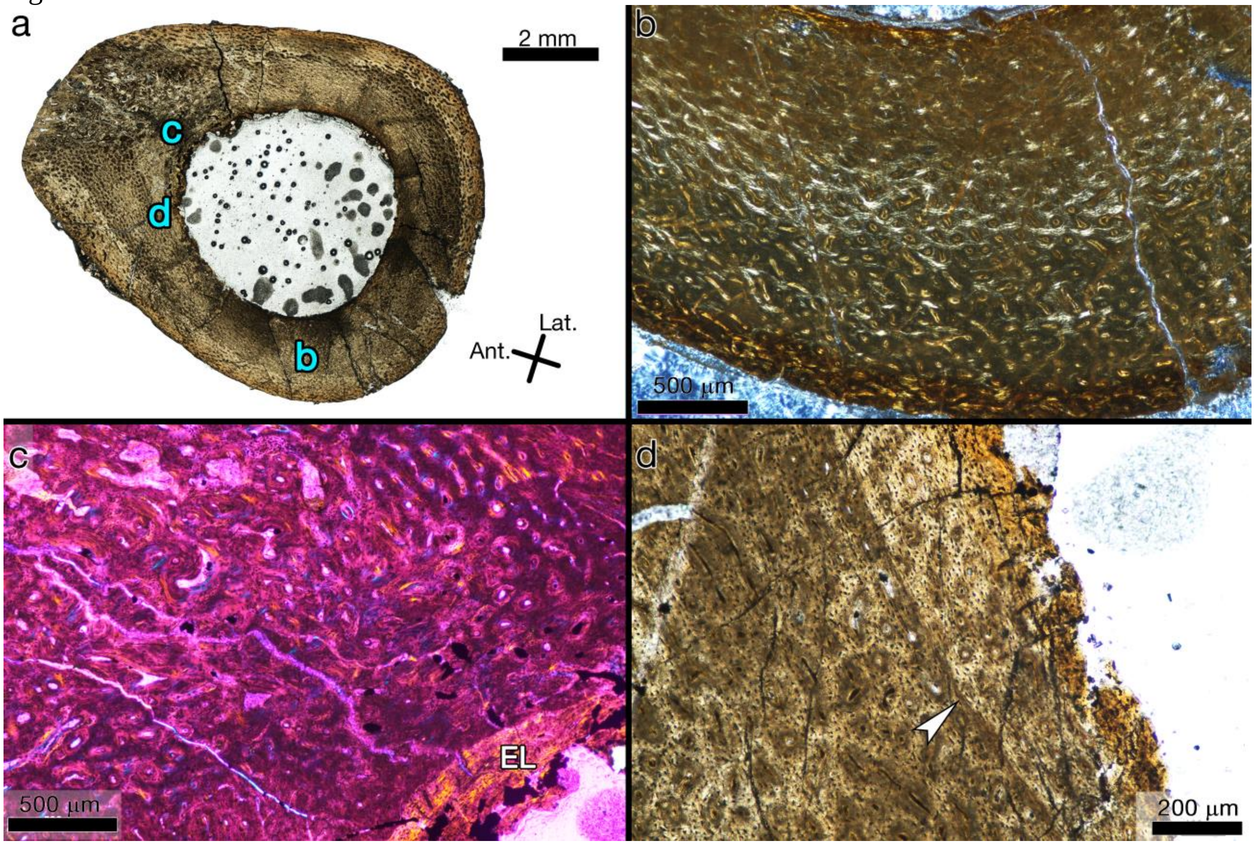

Supplementary Figure 6. Transverse section of right ornithopod tibia NMV P210062 MSC 602. a) Image of complete thin section. Blue letters reference the magnified regions shown in corresponding panels. Quarter wave plate. b) Tissue organization is fibro-lamellar to poorly organise parallel-fibred from inner to outer cortex. Primary osteons encircle longitudinal and reticular vascular canals. Circularly polarized light. c) Secondary osteons are dense and confined to the anterolateral plug. A thick endosteal lamellar layer is visible on the anterolateral side of the marrow cavity. EL = lamellar endosteal layer. Full wave plate. d) A LAG (arrow) is most visible on the anteromedial and anterolateral side of the inner cortex. Plane polarized light.

Figure S7.

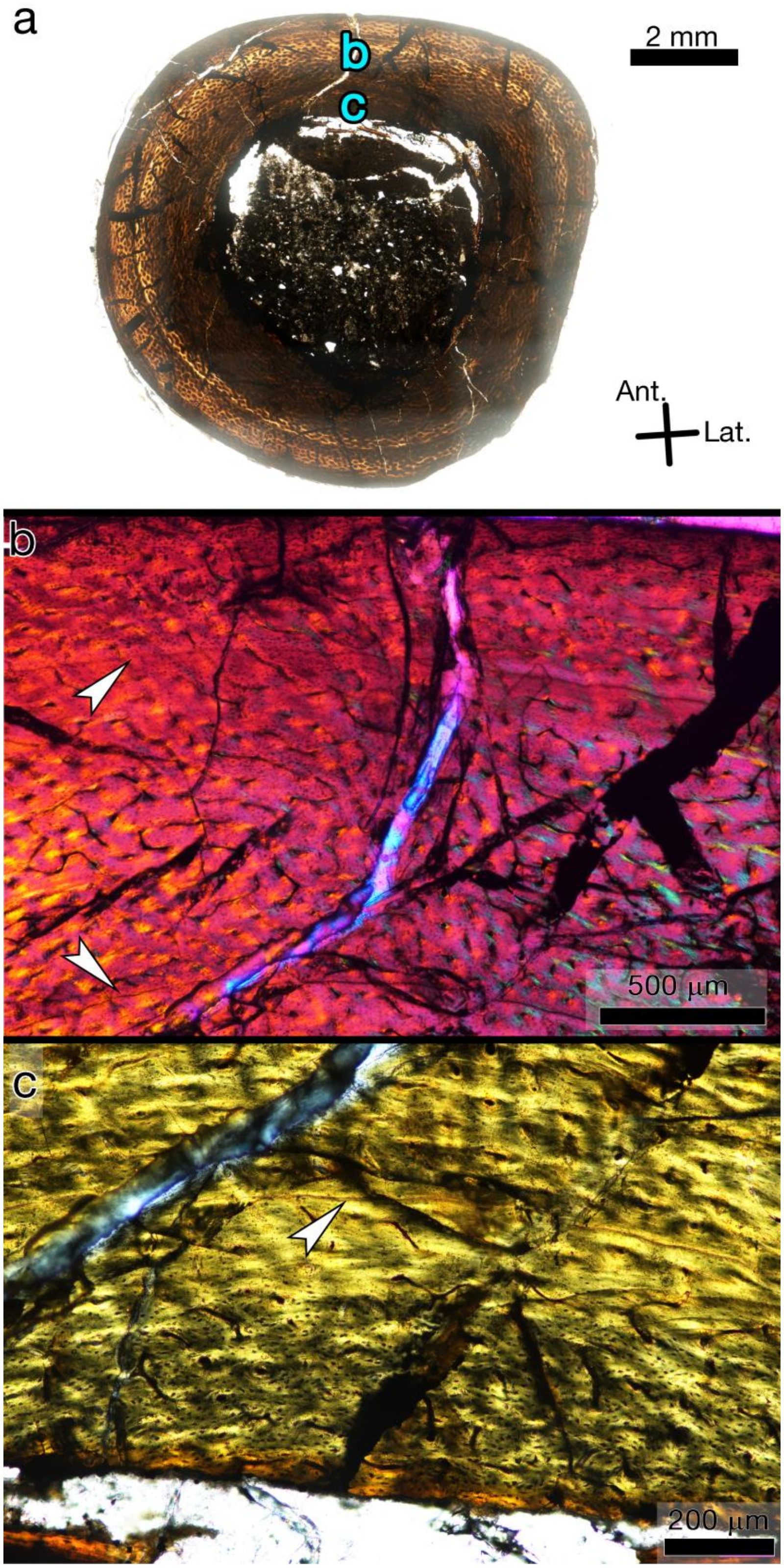

Supplementary Figure 7. Transverse section of right ornithopod tibia NMV P208204. a) Image of complete thin section. Opaque areas are the result of pyrite. Blue letters reference the magnified regions shown in corresponding panels. Plane polarized light. b) Two LAGs (arrows) are visible in the parallel-fibred cortex. Full wave plate. c) The innermost cortex on the anterior (shown) and posterior side is fibro-lamellar but changes to parallel-fibred after the first LAG. The cortex contains longitudinal and reticular simple vascular canals as well as small longitudinal primary osteons. The first of two LAGs (arrow) is visible within parallel-fibred tissue. Circularly polarized light.

Figure S8.

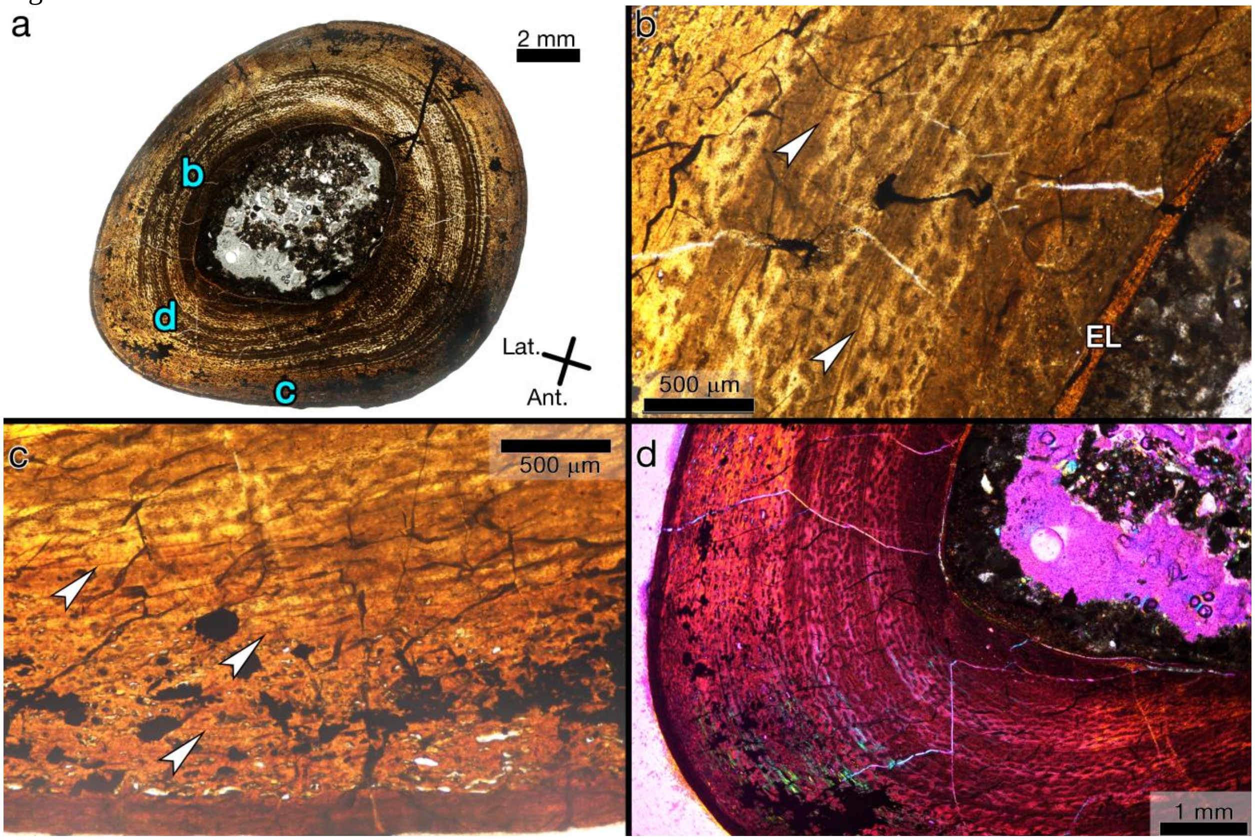

<u>Supplementary Figure 8</u>. Transverse section of left ornithopod tibia NMV P208336. a) Image of complete thin section. In many areas, pyrite has filled in vascular canals and taphonomic cracks in the bone. Blue letters reference the magnified regions shown in corresponding panels. Quarter wave plate. b) Preservation quality of the primary cortex is poor. However, a thin endosteal layer can be seen. The innermost of five LAGs (arrows) are also visible in this magnified region. EL = lamellar endosteal layer. Plane polarized light. c) The outermost three of five LAGs (arrows) are weakly visible within the mid-cortex despite the poor preservation of the specimen. Plane polarized light. d) The anterolateral region of the tibia enlarged to show pyrite invasion (opaque shapes within the cortex) as well as the parallel-fibred nature of the primary tissue. Full wave plate.

Figure S9.

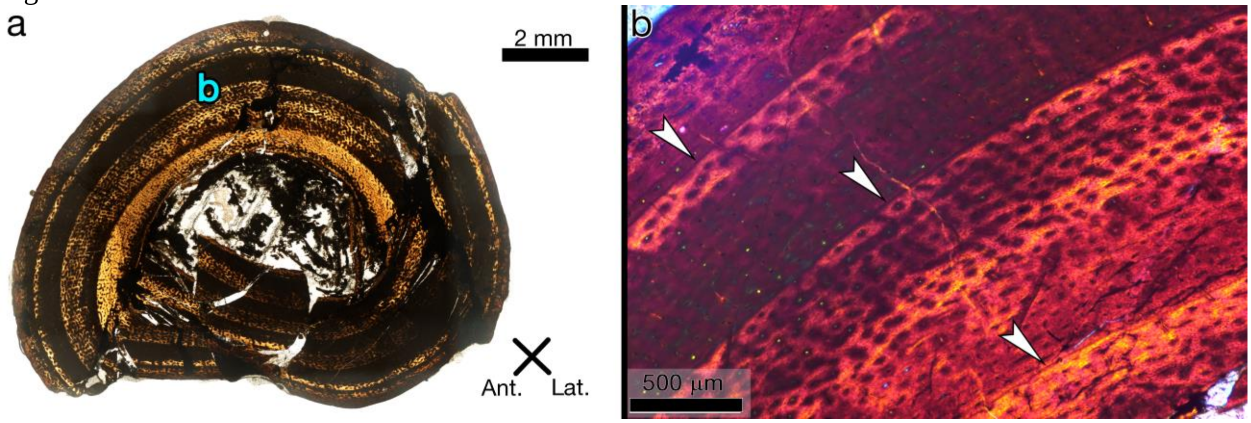

Supplementary Figure 9. Transverse section of right ornithopod tibia NMV P199133. a) Image of complete thin section. Opaque areas are the result of pyrite. Blue letter references the magnified region shown in the corresponding panel. Plane polarized light. b) Tissue is poorly preserved but appears to be parallel-fibred from inner to outermost cortex. Simple vascular canals are predominately longitudinal but reticular canals are scattered throughout the cortex. Three LAGs (arrows) are visible within the cortex. Opaque areas are the result of pyrite. Full wave plate.

Figure S10.

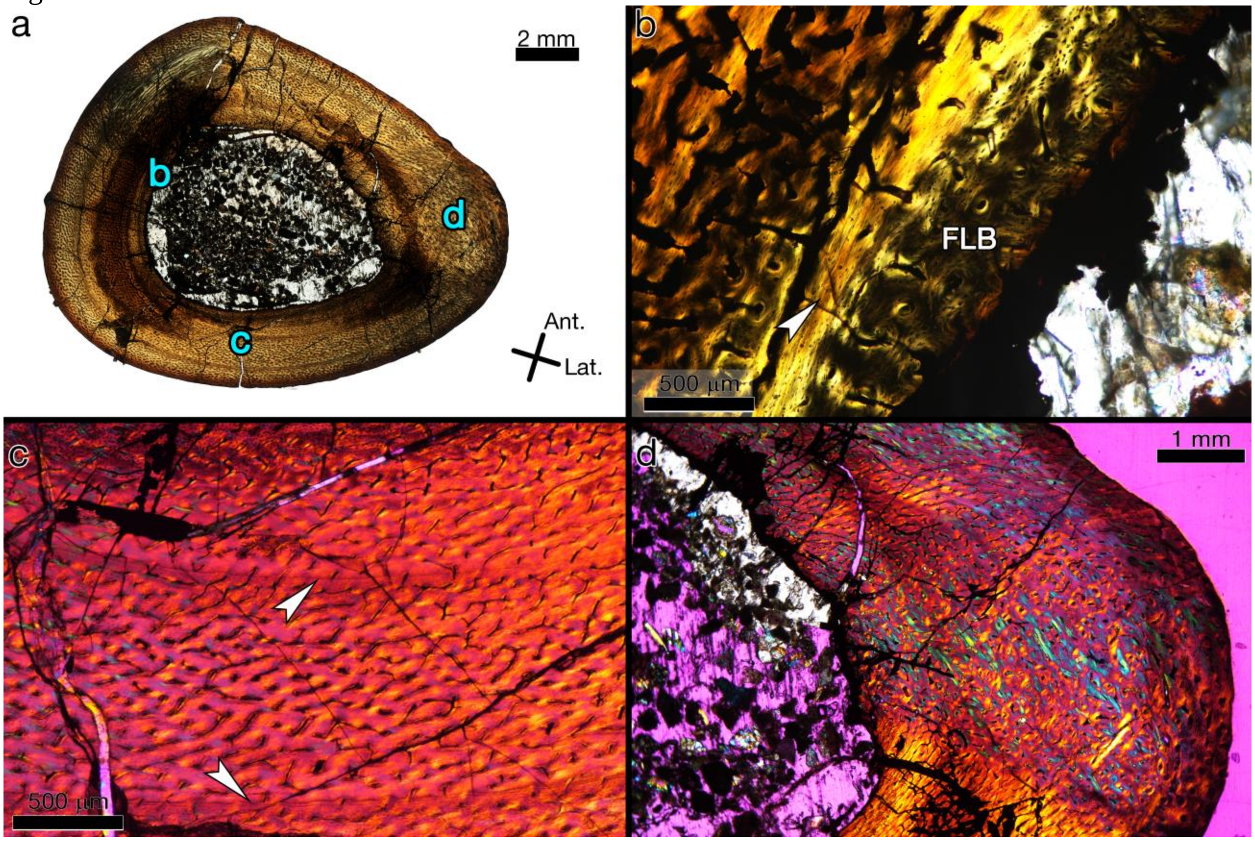

Supplementary Figure 10. Transverse section of right ornithopod tibia NMV P208189. a) Image of complete thin section. In many areas, pyrite has filled in vascular canals and taphonomic cracks in the bone. Blue letters reference the magnified regions shown in corresponding panels. b) The innermost cortex is longitudinal and reticular fibro-lamellar. In contrast, tissue from the middle to outer cortex is parallel-fibred with small primary osteons and simple primary vascular canals in anastomosing and reticular patterns. c) Two LAGs (arrows) are visible within the parallel-fibred cortex. Full wave plate. d) Secondary osteons are concentrated within the anterolateral plug region of the tibia transverse section. Full wave plate.

Figure S11.

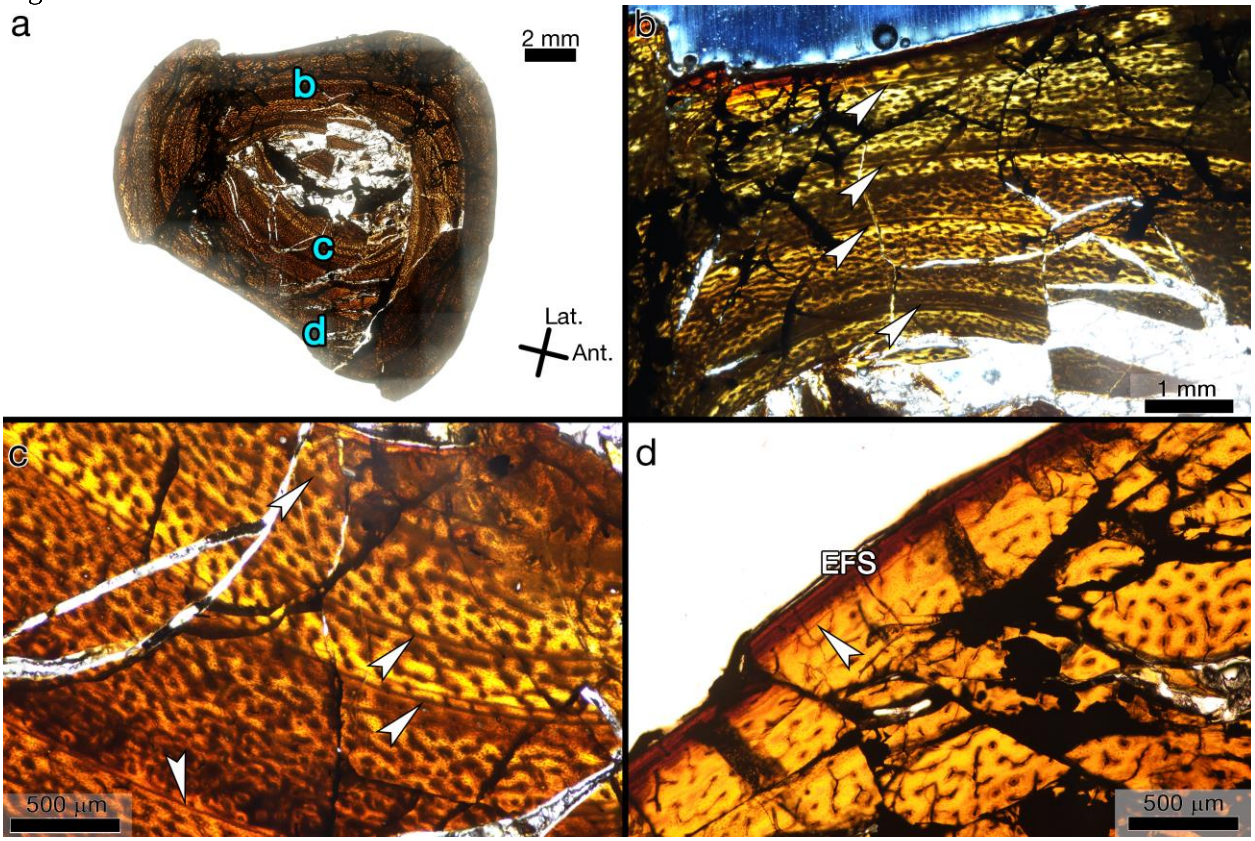

Supplementary Figure 11. Transverse section of left ornithopod tibia NMV P228434. The entire cortex is badly crushed and opaque regions indicate pyrite invasion into cracks. Blue letters reference the magnified regions shown in corresponding panels. Plane polarized light. b) The cortex is parallel-fibred with simple longitudinal or anastomosing vascular canals. Seven LAGs are visible within the cortex. In this panel, LAGs 3-6 (arrows) are visible but LAGs 1 and 2 were obliterated by medullary expansion. The seventh LAG is very close to the periosteal surface and not visible at this magnification. Circularly polarized light. c) The first four LAGs (arrows) are visible on the medial side. Plane polarized light. d) A magnified image of the outer cortex from the second of two serial thin sections. This second thin section was polished thinner than the first, to better reveal outermost cortical structures at the expense of inner cortical quality. The blue letter “d” in panel a) shows the region of interest from the second thin section. After the seventh LAG (arrow), vascularity greatly decreases within a possible EFS, containing an eighth LAG near the surface. Plane polarized light.

Figure S12.

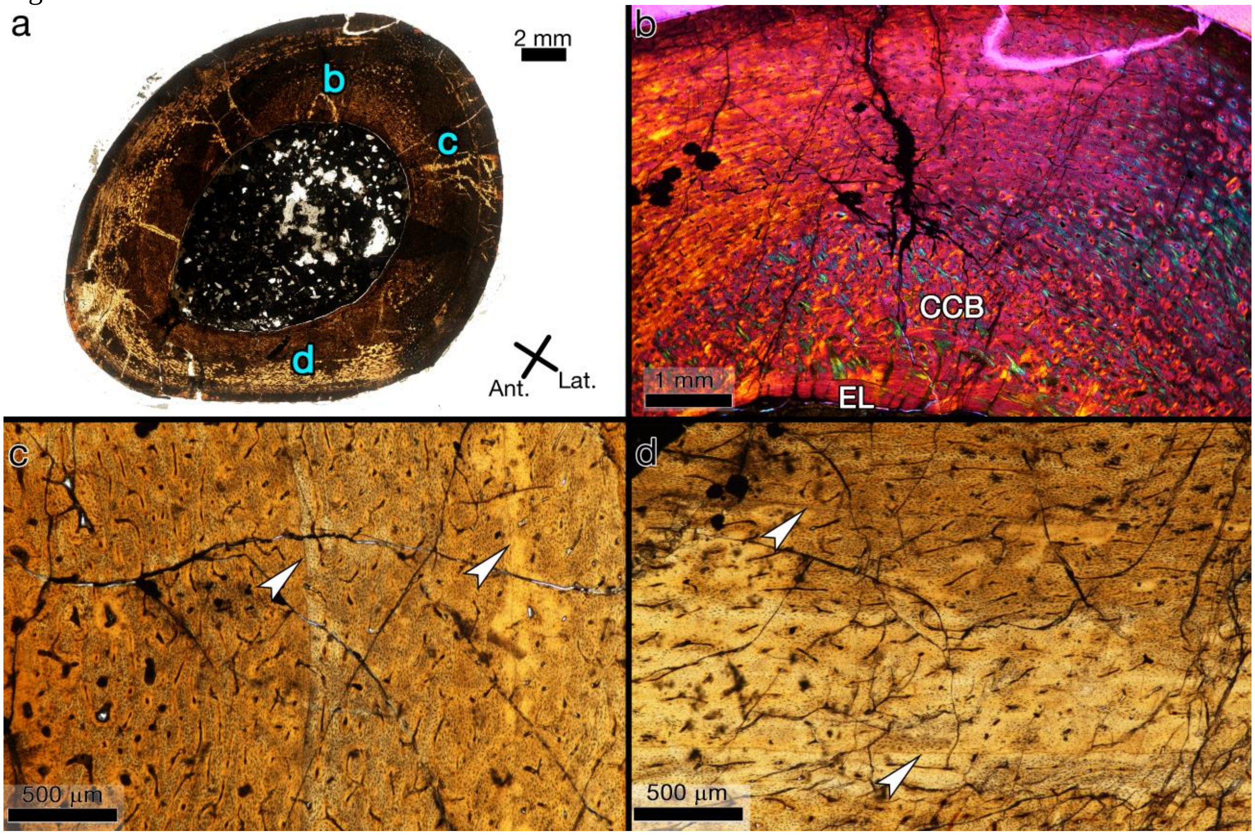

<u>Supplementary Figure 12.</u> Transverse section of left ornithopod femur NMV P186326. a) Composite image of complete thin section. Blue letters reference the magnified regions shown in corresponding panels. Plane polarized light. b) The innermost cortex on the posterior side consists of primary bone of endosteal origin, bounded by a thick lamellar endosteal layer. Compact coarse cancellous tissue and lamellar endosteal layer are traversed by radial channels. Full wave plate. CCB = compact coarse cancellous bone, EL = lamellar endosteal layer. c) Mid-cortex showing the first (innermost) and second LAG (arrows). Abundant osteocyte lacunae are embedded within primary cortex. Plane polarized light. d) Middle to outer cortex showing the second and third (outermost) LAG (arrows). Plane polarized light.

Figure S13.

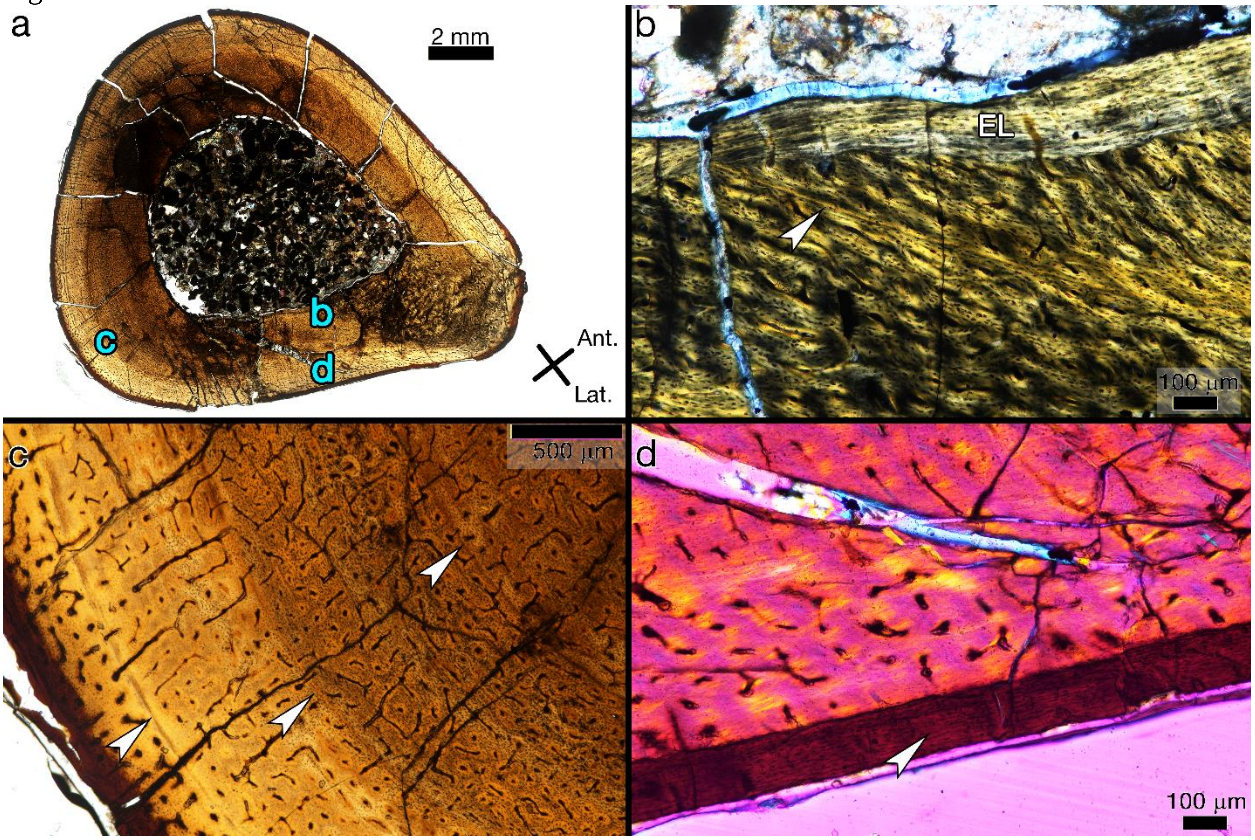

Supplementary Figure 13. Transverse thin section of right ornithopod tibia NMV P186334. a) Composite image of complete thin section, and showing a prominent anterior-lateral tibial border. Blue letters reference the magnified regions shown in corresponding panels. Quarter wave plate. b) The innermost cortex is fibro-lamellar and the medullary cavity is lined by a lamellar endosteal layer, which has within it radial channels. Arrow points to the innermost of four LAGs present within the cortex. This LAG is partially destroyed by medullary cavity enlargement. Circularly polarized light. EL = lamellar endosteal layer. c) The middle and outer cortex is primarily parallel-fibred and contains three LAGS (arrows). Plane polarized light. d) There is an abrupt band of colour change (arrow) in the outermost cortex, apparently due to mineral staining rather than a transition to EFS, as the tissue organization and vascular canal density remains constant across the boundary. Full wave plate.

Figure S14.

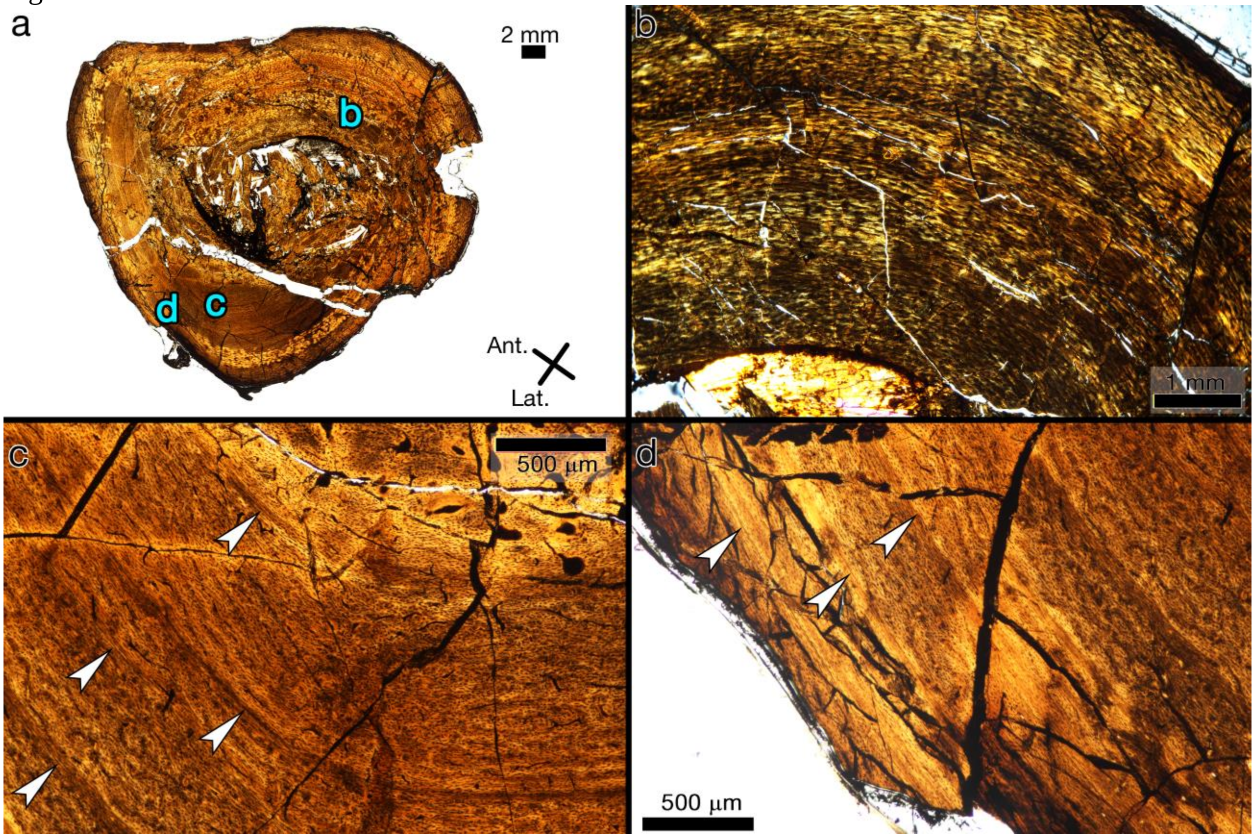

Supplementary Figure 14. Transverse section of right ornithopod tibia NMV P228360. Blue letters reference the magnified regions shown in corresponding panels. Plane polarized light. b) The primary tissue of the inner cortex varies between fibro-lamellar and poorly organized parallel-fibred, with longitudinal primary osteons, and is parallel-fibred from middle to outer cortex with longitudinal primary osteons and anastomosing simple vascular canals. Circularly polarized light. c) Four annuli are present within the inner to middle cortex. The innermost annulus is partially destroyed by medullary expansion. Plane polarized light. d) Three annuli are present within the outer cortex, for a total of seven annuli. Plane polarized light.

Supplementary Information Table 1. A summary of bone fibre orientation, vascular canal orientation, and associated apposition rates reported from the literature, which generally supports 'Amprino's Rule'.

**Fibre Orientation**	**Vascular Canal Orientation**	**Apposition rate (μm/day)**	**Reference**
Lamellar		<1-1	1
Parallel-fibred		1	1
Fibrolamellar			
	Reticular	10-39.5	1, 2
	Laminar	10-39.5	1, 2
	Sub Plexiform	10-39.5	1, 2
	Longitudinal	4.8-50	2, 3
	Plexiform	5-15	1
Woven		5-171	1-6

References

1. Castanet, J., Grandin, A., Abourachid, A. & de Ricqlès, A. Expression de la dynamique de croissance dans la structure de l'os périostique chez Anas plathyrhynchos. Comptes Rendus de l'Academie des Sciences Serie III Sciences de la Vie 319, 301-308 (1996).

2. Castanet, J., Rogers, K. C., Cubo, J. & Boisard, J.-J. Periosteal bone growth rates in extant ratites (ostriche and emu). Implications for assessing growth in dinosaurs. Comptes Rendus de l'Academie des Sciences Serie III Sciences de la Vie 323, 543-550 (2000).

3. Starck, J. M. & Chinsamy, A. Bone microstructure and developmental plasticity in birds and other dinosaurs. Journal of Morphology 254, 232-246 (2002).

4. de Margerie, E. et al. Assessing a relationship between bone microstructure and growth rate: a flourescent labelling study in the king penguin chick (Aptenodytes patagonicus). Journal of Experimental Biology 207, 869-879 (2004).

5. de Margerie, E. Laminar bone as an adaptation to torsional loads in flapping flight. Journal of Anatomy 201, 521-526 (2002).

6. de Margerie, E., Cubo, J. & Castanet, J. Bone typology and growth rate: testing and quantifying 'Amprino's rule' in the mallard (Anas platyrhynchos). Comptes Rendus Biologies 325, 221-230 (2002).

Supplementary Information Table 2. Quantitative measurements for NMV femora and tibiae. CGM Count refers to cyclical growth marks, numbered consecutively from innermost to outermost cortex. Circumference refers to the perimeter measurement of either medullary cavity, CGMs, or periosteal surface. Inclusive Area refers to the entire area within the circumferential outline. Zonal Area is the cortical area between CGMs. Total Cortical Area is the area of the entire cortex, which excludes the medullary cavity. Major and minor axis diameters were calculated for each circumferential measurement.

**Locality**	**NMV Specimen**	**CGM Count**	**Circumference (cm)**	**Inclusive Area (cm^2)**	**Zonal Area (cm^2)**	**Total Cortical Area (cm^2)**	**Major Axis Diameter (cm)**	**Minor Axis Diameter (cm)**
Flat Rocks	P216768	Medullary Cavity	1.0	0.1			0.3	0.2
		Surface	1.8	0.2	0.2	0.2	0.6	0.4

**Locality**	**NMV Specimen**	**CGM Count**	**Circumference (cm)**	**Inclusive Area (cm^2)**	**Zonal Area (cm^2)**	**Total Cortical Area (cm^2)**	**Major Axis Diameter (cm)**	**Minor Axis Diameter (cm)**
Flat Rocks	P208495	Medullary Cavity	2.3	0.4			0.8	0.6
		1	3.1	0.6	0.3		1.0	0.8
		2	Too incomplete					
		Surface	3.5	0.9		0.5	1.2	1.0

**Locality**	**NMV Specimen**	**CGM Count**	**Circumference (cm)**	**Inclusive Area (cm^2)**	**Zonal Area (cm^2)**	**Total Cortical Area (cm^2)**	**Major Axis Diameter (cm)**	**Minor Axis Diameter (cm)**
	P199058							
Flat Rocks		CGM Count	Perimeter (cm)	Inclusive Area (cm^2)	Zonal Area (cm^2)	Total Cortical Area (cm^2)	Major Axis Diameter (cm)	Minimum Axis Diameter (cm)

		Medullary Cavity	2.8	0.5			0.9	0.7
		1	Too incomplete					
		2	Too incomplete					
		3	Too incomplete					
		Surface	4.7	1.4		0.9	1.5	1.2

Locality	NMV Specimen	CGM Count	Circumference (cm)	Inclusive Area (cm^2)	Zonal Area (cm^2)	Total Cortical Area (cm^2)	Major Axis Diameter (cm)	Minor Axis Diameter (cm)
Flat Rocks	P221151	Medullary Cavity	3.3	0.7			1.1	0.9
		1	3.0	0.6	-0.1		0.8	0.9
		2	3.5	0.9	0.3		1.0	1.0
		3	4.0	1.1	0.3		1.2	1.2
		4	4.6	1.4	0.3		1.4	1.3
		5	5.1	1.8	0.4		1.6	1.4
		Surface	5.5	2.0	0.3	1.3	1.7	1.5

Locality	NMV Specimen	CGM Count	Circumference (cm)	Inclusive Area (cm^2)	Zonal Area (cm^2)	Total Cortical Area (cm^2)	Major Axis Diameter (cm)	Minor Axis Diameter (cm)
Flat Rocks	P210062	Medullary Cavity	1.5	0.2			1.0	0.4
		1	1.7	0.2	0.0		1.1	0.4
		Surface	3.0	0.6	0.4	0.4	1.5	0.7

Locality	NMV Specimen	CGM Count	Circumference (cm)	Inclusive Area (cm^2)	Zonal Area (cm^2)	Total Cortical Area (cm^2)	Major Axis Diameter (cm)	Minor Axis Diameter (cm)

Flat Rocks	P208204	Medullary Cavity		1.7	0.2			0.5	0.5
			1	1.9	0.2			0.6	0.6
			2	2.7	0.5			0.8	0.8
		Surface		3.1	0.7		0.5	1.0	0.9

Locality	NMV Specimen	CGM Count		Circumference (cm)	Inclusive Area (cm^2)	Zonal Area (cm^2)	Total Cortical Area (cm^2)	Major Axis Diameter (cm)	Minor Axis Diameter (cm)
Flat Rocks	P208336	Medullary Cavity		2.0	0.3			0.7	0.5
			1	2.5	0.4	0.1		0.8	0.7
			2	3.1	0.7	0.2		1.0	0.9
			3	3.6	0.9	0.2		1.2	1.0
			4	3.9	1.0	0.2		1.3	1.1
			5	4.2	1.2	0.2		1.4	1.1
		Surface		4.5	1.4	0.2	1.1	1.5	1.2

Locality	NMV Specimen	CGM Count		Circumference (cm)	Inclusive Area (cm^2)	Zonal Area (cm^2)	Total Cortical Area (cm^2)	Major Axis Diameter (cm)	Minor Axis Diameter (cm)
Flat Rocks	P199133	Medullary Cavity		1.9	0.1			0.5	0.3
			1	2.6	0.3	0.2		0.7	0.4
			2	3.4	0.5	0.2		1.0	0.6
			3	3.9	0.7	0.2		1.1	0.7
		Surface		3.9	0.9	0.2	0.8	1.2	0.9

Locality	NMV Specimen	CGM Count	Circumference (cm)	Inclusive Area (cm^2)	Zonal Area (cm^2)	Total Cortical Area (cm^2)	Major Axis Diameter (cm)	Minor Axis Diameter (cm)
Flat Rocks	P208189	Medullary Cavity	2.3	0.3			0.7	0.6
		1	2.9	0.5	0.2		1.0	0.7
		2	3.9	1.0	0.4		1.3	1.0
		Surface	4.4	1.3	0.3	1.0	1.5	1.1

Locality	NMV Specimen	CGM Count	Circumference (cm)	Inclusive Area (cm^2)	Zonal Area (cm^2)	Total Cortical Area (cm^2)	Major Axis Diameter (cm)	Minor Axis Diameter (cm)
Flat Rocks	P228434	Medullary Cavity	2.0	0.2			0.4	0.4
		1	Too incomplete					
		2	2.3	0.2			0.7	0.4
		3	2.7	0.3	0.1		0.8	0.4
		4	3.7	0.6	0.3		1.1	0.7
		5	4.3	0.9	0.3		1.3	0.9
		6	5.0	1.4	0.5		1.5	1.1
		7	5.2	1.6	0.2		1.5	1.2
		8	Too incomplete					
		Surface	5.2	1.6	0.1	1.5	1.6	1.3

Locality	NMV Specimen	CGM Count	Circumference (cm)	Inclusive Area (cm^2)	Zonal Area (cm^2)	Total Cortical Area (cm^2)	Major Axis Diameter (cm)	Minor Axis Diameter (cm)
Dinosaur Cove	P180892	Medullary Cavity	5.2	1.5			1.4	1.7

		1	Too remodelled to trace					
		2	Too remodelled to trace					
		3	Too remodelled to trace					
		4	Too remodelled to trace					
		5	Too remodelled to trace					
		6	Too remodelled to trace					
		7	Too remodelled to trace					
		Surface	10.1	6.8	5.3	5.3	3.2	2.9

**Locality**	**NMV Specimen**	**CGM Count**	**Circumference (cm)**	**Inclusive Area (cm^2)**	**Zonal Area (cm^2)**	**Total Cortical Area (cm^2)**	**Major Axis Diameter (cm)**	**Minor Axis Diameter (cm)**
Dinosaur Cove	P177935	Medullary Cavity	4.3	1.3			1.4	1.2
		1	Not present					
		2	Not present					
		3	4.8	1.6	0.3		1.4	1.3
		4	6.1	2.5	0.9		1.9	1.6
		5	6.8	3.1	0.6		2.2	1.7
		6	7.1	3.4	0.3		2.3	1.8
		7	7.4	3.6	0.2		2.3	1.9
		Surface	7.5	3.7	0.1	2.4	2.3	2.0

Locality	NMV Specimen	CGM Count	Circumference (cm)	Inclusive Area (cm^2)	Zonal Area (cm^2)	Total Cortical Area (cm^2)	Major Axis Diameter (cm)	Minor Axis Diameter (cm)
Dinosaur Cove	P186326	Medullary Cavity	3.4	1.0			1.0	0.9
		1	Not present					
		2	4.1	1.2			1.5	1.1
		3	5.2	1.9			1.7	1.4
		4	5.9	2.4			2.0	1.6
		Surface	6.4	2.8		1.9	2.2	1.7

Locality	NMV Specimen	CGM Count	Circumference (cm)	Inclusive Area (cm^2)	Zonal Area (cm^2)	Total Cortical Area (cm^2)	Major Axis Diameter (cm)	Minor Axis Diameter (cm)
Dinosaur Cove	P228360	Medullary Cavity	2.4	0.4			0.8	0.6
		1	Too incomplete					
		2	3.4	0.8	0.4		1.1	0.9
		3	3.9	1.0	1.0		1.3	1.0
		4	4.3	1.2	0.5		1.4	1.2
		Surface	4.5	1.3	0.4	1.0	1.5	1.2
